# Wideband metamaterial perfect absorber using topological insulator material for infrared and visible light spectrum: a numerical approach

**DOI:** 10.1038/s41598-025-14623-7

**Published:** 2025-08-05

**Authors:** Vishal Sorathiya, Zen A. Sbeah, Ahmad Alghamdi, Amar Y. Jaffar, Abdullah G. Alharbi

**Affiliations:** 1 Faculty of Engineering and Technology, Parul Institute of Engineering and Technology, Parul University, Waghodia Road, Vadodara, 391760 Gujarat India; 2 Department of Mechanical and Industrial Engineering, College of Engineering and Computing in Al-Qunfudhah, Umm al-Qura University, Mecca, Saudi Arabia; 3 Computer and Network Engineering Department, College of Computing, Umm Al-Qura University, Makkah, Saudi Arabia; 4 Department of Electrical Engineering, College of Engineering, Princess Nourah bint Abdulrahman University, P.O. Box 84428, Riyadh, 11671 Saudi Arabia

**Keywords:** Solar cell absorber, Topological insulator (TI), Wideband absorber, Metamaterial absorbers, Metamaterials, Nanophotonics and plasmonics

## Abstract

This study utilises simulations to investigate the potential of a novel multi-layered topological insulator-based wideband absorber design. The proposed design is constructed with a multilayer structure that incorporates meticulously chosen materials to enhance light absorption. The top layer is composed of a metal (Fe/Ti/Cu/Zn/Ag/Au), which is followed by an insulating layer (Si/SiO₂/InP) and a topological insulator (Bi₁.₅Sb₀.₅Te₁.₈Se₁.₂). These layers are sandwiched between two metal layers (Fe/Ti/Cu/Zn/Ag/Au). The proposed structure is analysed for two different resonator-based designs, considering both the L-shaped metal resonator and the complementary L-shaped resonator for the overall computational analysis. The overall structure is computed for the broad range of the wavelength spectrum (0.2–1.6 μm). The proposed metamaterial design achieves an absorption rate of ~ 99% across multiple wavelength bands. This structure also investigated the different parametric values, such as physical dimensions and oblique angle of incident, to identify the optimised values of the different parameters. The metamaterial parameters, such as permittivity, permeability, refractive index, and impedance values, are also investigated over the entire wavelength spectrum, which suggests that the overall structure behaves as a double negative material. The wideband metamaterial structure with topological insulator material can also be compared with the interference mode theory.

## Introduction

The development of advanced materials that mitigate the detrimental effects of conventional energy sources is essential for the construction of a more sustainable and environmentally friendly energy future. It is essential to identify the suitable materials that minimise the environmental impact of renewable energy technologies, such as energy storage systems, wind turbines, and solar cells, while also maximising their efficiency. By pushing the limits of material science, we can create a future where clean energy is not only plentiful but also genuinely environmentally sustainable^1,2^. Traditional energy sources have a major negative impact on the environment and exacerbate climate change. The need for new material-related research is growing, as it requires creative studies of new materials to aid in the creation of technologies for renewable energy^3^. Traditional energy sources can address these challenges, but metamaterial plasmonic absorbers offer a revolutionary approach. By using engineered nanostructures to control light at the nanoscale, these absorbers achieve exceptional sensitivity^4^. These types of absorbers work by inducing surface plasmons, which produce angular resonance peaks that precisely adjust to even the smallest changes in their surroundings. These types of structures are helpful for complex optical and energy applications because of this unique capability^5,6^. Researchers can significantly increase their absorbing parameters by optimising the design of these metamaterials. These types of researchers open up a range of applications, like environmental monitoring and biomolecule detection^7–10^.

The field of solar energy is undergoing a significant transformation due to the emergence of metamaterials, which are nanoscale-designed, artificially structured materials. These materials can be precisely engineered to achieve exceptional light absorption. By carefully controlling the interaction between light and matter, they allow for the creation of solar absorbers that outperform traditional designs and capture almost all incoming light across a broad spectrum of wavelengths^11–14^. Consequently, this leads to a substantial increase in energy conversion efficiency, facilitating the development of advanced solar technologies that minimise environmental impact. The capacity to manipulate light at this fundamental level positions metamaterials as a critical element in the advancement of future solar energy systems. The capacity to precisely engineer electromagnetic resonances, spanning both localised and propagating wave regions, shows these materials as immensely valuable for energy technologies across the frequency spectrum, from low to high^15^. This technology demonstrates significant promise in boosting the efficiency of solar thermal energy harvesters, especially those operating at medium to high temperatures^16,17^.

In recent years, topological insulators (TIs) have attracted significant attention within the scientific community. These enigmatic particles have captured global interest, necessitating the development of intricate devices incorporating TIs. The pioneering discovery and characterisation of the first two-dimensional TI, HgTe, in 2007 set the stage for further advances^18^. Several three-dimensional materials, including Bi₂Se₃, Bi₂Te₃, and Sb₂Te₃, have been identified by researchers as TIs. Although these materials are highly conductive on their surfaces, they also behave as insulators in bulk. They have become the primary focus of current condensed matter research because of this unique feature. It has remarkable electronic properties that make it possible to develop spintronic applications because of its topologically protected surface states. This material can be applied to various quantum computing and next-generation electronics applications. These materials are further improved by their resistance to impurities and defects, which helps to lower energy loss and improve device performance. The discovery of composited TI materials has led to a notable increase in interest in utilising materials such as HgTe, Bi₂Se₃, and Bi₂Te₃ to their full potential^19^. The growing interest in TIs is a sign of both their theoretical and practical potential, as scientists work to integrate them into advanced technologies. An excellent illustration of an engineered TI designed to maximise surface conduction while minimising bulk interference is Bi₁.₅Sb₀.₅Te₁.₈Se₁.₂^20^. An innovative method of enhancing electromagnetic absorption is the incorporation of TIs into metamaterial absorbers^21^. The surface properties facilitate the development of resonant structures with robust, broad-spectrum absorption when they are incorporated into metamaterial designs^22^. Bi₁.₅Sb₀.₅Te₁.₈Se₁.₂/Topological insulator (TI) offers several quantitative advantages over conventional semiconductors in terms of photonics absorption, primarily due to its unique plasmonic and optoelectronic properties. TI is identified as an effective plasmonic material in the visible and near-infrared range, capable of confining approximately 80% of electromagnetic field energy at the resonance peak, which is significantly higher than many traditional semiconductors^23^. This strong plasmonic resonance is crucial for controlling electromagnetic waves, making BSTS highly suitable for advanced optoelectronic devices. Additionally, TI’s ability to support higher-order resonance peaks further enhances its utility in photonics applications^23^. In comparison, other materials like Bi₂Te₂S and Bi₂Te₂Se, while having high electron mobilities and moderate band gaps, do not exhibit the same level of plasmonic resonance, although they are efficient in absorbing sunlight across the solar spectrum^24^. Furthermore, the Te/Bi₂Se₃ heterojunction demonstrates a broad photodetection range and high responsivity, but it is primarily noted for its broadband detection capabilities rather than specific plasmonic advantages^25^. Overall, the high electromagnetic field confinement and resonance capabilities of BSTS provide it with a distinct edge in photonics absorption over conventional semiconductors, making it a promising candidate for future optoelectronic applications.

Broadband absorption is essential for energy harvesting applications, for both photovoltaic and solar-thermal systems, capturing the widest possible portion of the solar spectrum is fundamental to maximising conversion efficiency, including solar thermal and photo detectors. Covering both IR and visible ranges ensures maximum solar spectrum utilisation, improving device efficiency. By combining plasmonic metals (Ag/Au/Ti/Cu) with dielectric spacers (SiO₂/Si/InP) and materials like TIs (Bi₁.₅Sb₀.₅Te₁.₈Se₁.₂), one can engineer overlapping resonances like SPR, FP cavity and topological surface states. Materials must be chosen to minimise parasitic losses outside of the desired absorption. TIs may offer lower losses in some IR regimes compared to conventional metals. The goal is to achieve more than 90% absorption over a specified continuous wavelength range. The broader the range, the more challenging the overlap of resonances, but also the greater the performance.

This paper introduces a novel multi-layered absorber design that is engineered for wideband absorption and is based on TIs. It introduces two distinct designs: Design M1, which includes an L-shaped resonator, and Design M2, which includes a complementary L-shaped resonator. Both are designed to effectively absorb light across a wide range of wavelengths (0.2 to 1.6 μm) spectrum, including ultraviolet (UV), visible (VIS), and near-infrared (NIR) light. The proposed absorbers are numerically investigated for different physical parameters to identify the optimised performance. This paper also presented the calculation of effective optical parameters for the entire wavelength spectrum to identify the effect of the metamaterial behaviour.

### Modelling and design

**Fig. 1:**
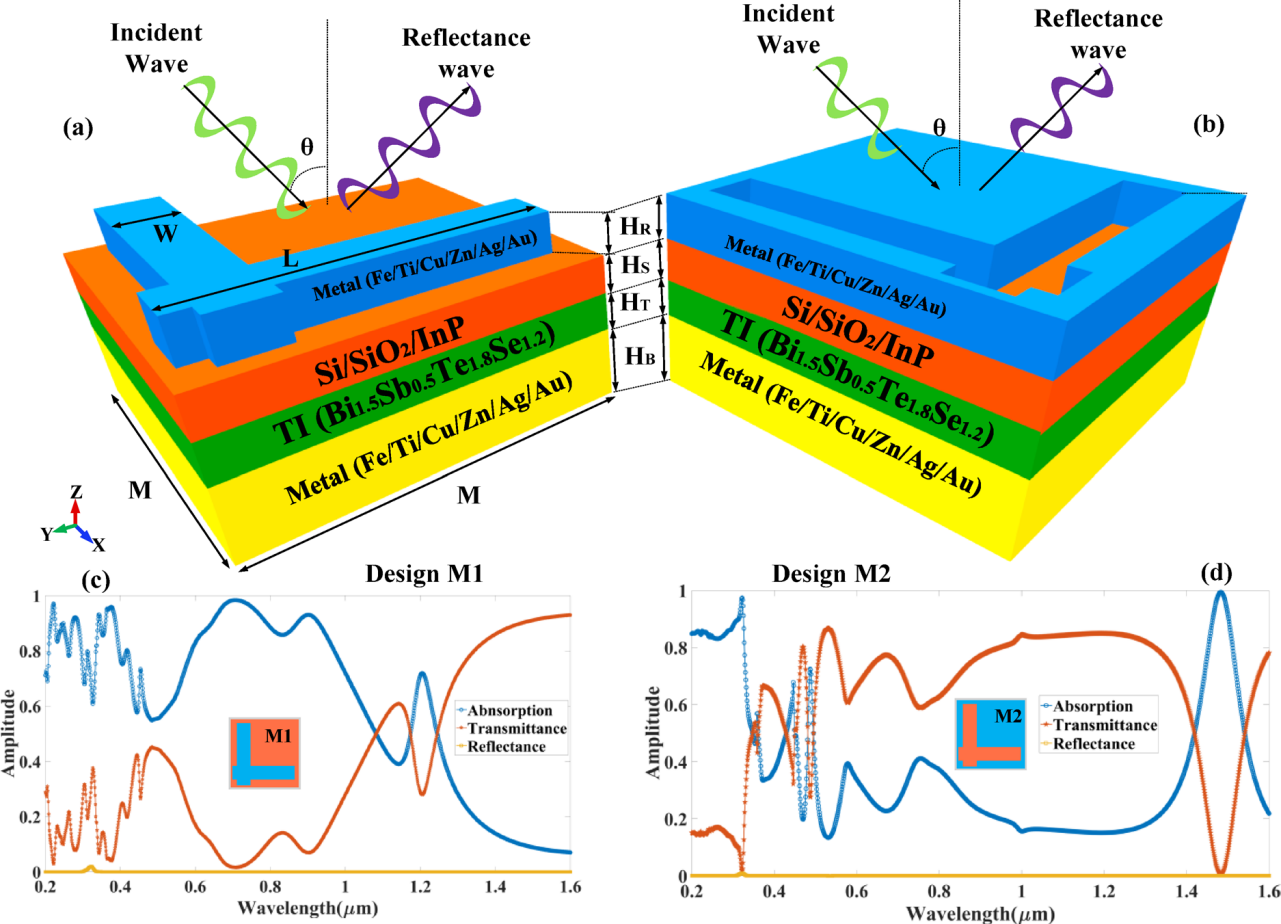
Schematic of the multi-layered TI (Bi_1.5_Sb_0.5_Te_1.8_Se_1.2_) based wideband absorber for the different (**a**) L resonator structure and (**b**) complementary L resonator design. The absorber structure is formed with the layers of the bottom metal (Fe/Ti/Cu/Zn/Ag/Au), TiO2 (Si/SiO2/InP), and resonator metal shape (Fe/Ti/Cu/Zn/Ag/Au). The variation in the absorption, reflectance and transmittance for the different shapes of resonator (**c**) Design M1 and (**d**) Design M2 structure. The incident wave for the excitation of a broad wavelength spectrum (0.2 to 1.6 μm) is launched from the top of the structure. The variation in the resonance band is generated for the different wavelength bands for both structures.

A wideband absorber based on a multi-layered TI is depicted in Fig. 1. COMSOL Multiphysics software is employed to develop this structure. The top port is regarded as an incident light source from the sun. In order to identify the transmitted wave, the bottom layer is regarded as the second port. The proposed two-port structure is used to determine the absolute values of transmittance, reflectance, and absorption by calculating the variation in the S parameters. A periodic boundary condition is defined in the X-Y direction for the proposed two-port network as shown in Fig. 1(a). Maximum meshing size is 24 nm, and minimum meshing size is 0.24 nm. The curvature factor is set to 0.2, and the maximum element growth rate is 1.3 for triangular meshing conditions. The two designs, Design M1 and Design M2, are numerically analysed to determine their overall structure. Design M1 includes an L-shaped resonator, while Design M2 includes a complementary L-shaped resonator. Both designs are optimised to achieve broadband absorption across the wavelength range of 0.2 μm to 1.6 μm, which encompasses ultraviolet (UV), visible (VIS), and near-infrared (NIR) light. The incident wave in launched from the top of the structure in TE and TM mode of the polarization as shown in Fig. 1. Additionally, Fig. 1 illustrates a metallic ground plane (Fe/Ti/Cu/Zn/Ag/Au) that functions as a reflective layer, thereby preventing wave transmission and guaranteeing the complete confinement of energy within the structure. Above this, the electromagnetic interactions are further augmented by the strong surface plasmon resonance properties of a TI (Bi₁.₅Sb₀.₅Te₁.₈Se₁.₂). Impedance matching is facilitated by a dielectric interlayer (Si/SiO₂/InP), which minimises reflection losses and supports resonant excitation for maximum absorption. The resonator metal structure, which is composed of the same metal as the base, is present in the uppermost layer. It is designed in two configurations for Design M1 and Design M2. These two configurations enable selective resonant modes by modifying the current distribution and localised field enhancement, directly impacting the broadband absorption performance. The refractive index of different material Fe^26^, Ti^27^, Cu^26^, Zn^27^, Ag^26^, Au^26^, Si^28^, SiO₂^28^, InP and Bi₁.₅Sb₀.₅Te₁.₈Se₁.₂^29^ is considered from its experimental datas from different depositories.

SPR plays a dominant role in enhancing localised absorption at the metal–dielectric interface, especially in the visible range (0.2–0.6 μm). This effect is evident in the electric field distribution plots, Figs. 9(a-h) and 10(a-h) for both Mode M1 and Mode M2 designs, where strong field confinement is observed near the resonator edges. These localised resonances arise from collective oscillations of conduction electrons, characteristic of SPR. The multi-layered MIM cavity formed between the top resonator and bottom metallic reflector acts as a Fabry–Pérot resonator. Multiple internal reflections and constructive interference contribute to wideband absorption. To support this, we compared the analytically calculated absorption using interference theory with simulated absorption as shown in Figs. 7(e) and 8(e). The strong agreement between both confirms the role of Fabry–Pérot resonance in the design. Near-field interactions occur due to strong electromagnetic coupling between adjacent layers and resonators, especially at higher wavelengths and oblique incidence. It leads to hybrid modes that broaden and intensify the absorption response. The magnetic field distribution plots, Figs. 9(i-p) and 10(i-p), show deep field penetration and localised enhancement within the dielectric and metallic layers, supporting the presence of strong near-field coupling^30,31^. The significance of these structures lies in their ability to support multi-resonant absorption bands due to the interplay of surface plasmon resonances (SPRs), Fabry-Pérot interference, and near-field coupling effects, all of which contribute to enhanced absorption efficiency over a wide spectral range. The incident electromagnetic wave, depicted in the schematic, is launched from the top of the absorber at an oblique angle (θ), interacting with the layered structure to generate strong resonant absorption. Design M1 and Design M2, as illustrated in Figs. 1 (c) and 1 (d), exhibit absorption behaviour that is determined by their unique resonator geometries. The characteristics of the absorber can be optimised by adjusting the geometric parameters of each layer, including its width (W), length (L), and thickness (H). The dielectric layer in the depicted structure, particularly the topological insulator (TI) layer, plays a critical role in enhancing optical absorption and tailoring the resonance characteristics of the device. This layer, when sandwiched between metal layers and a semiconductor substrate (Si/SiO₂/InP), acts as a low-loss medium that supports strong electromagnetic confinement through interference and plasmonic coupling mechanisms. It facilitates the formation of hybrid modes such as gap surface plasmon resonances and dielectric waveguide resonances, which enhance light–matter interaction over a broad spectral range. As seen in the absorption spectra, Fig. 1(c) and (d), the dielectric layer significantly influences the absorption peak positions and bandwidths in both M1 and M2 designs, allowing for tunable spectral selectivity and higher absorption efficiencies, particularly in the near-infrared range. Moreover, the presence of the TI layer introduces topologically protected surface states, which contribute to reduced backscattering and enhanced robustness under oblique incidence, thereby improving the overall performance of the absorber in photodetection or photovoltaic applications. Table 1 shows the optimised parameters selected for the overall structure. In all parametric variations of the results, the single values of each parameter change, while other parameters remain constant for the overall computational analysis.

**Table 1:** Values of the different optimised parameters of the proposed absorber structure.

M	HB	HS	HR	L	W	θ
500 nm	100 nm	100 nm	50 nm	300 nm	125 nm	0°

## Results and discussion

Phase matching is a critical condition for efficiently exciting surface plasmons (SPs) at a metal-dielectric interface. It ensures momentum conservation between the incident light and the plasmonic mode. Phase matching requires that the wavevector (momentum) of the incident light matches that of the surface plasmon polariton (SPP) at the interface^32^:

1 $$\:{\varvec{k}}_{\varvec{S}\varvec{P}\varvec{P}}={\varvec{k}}_{\varvec{i}\varvec{n}\varvec{c}\varvec{i}\varvec{d}\varvec{e}\varvec{n}\varvec{t}}$$

Where: $\:{\varvec{k}}_{\varvec{S}\varvec{P}\varvec{P}}=$ wavevector of the surface plasmon. $\:{\varvec{k}}_{\varvec{i}\varvec{n}\varvec{c}\varvec{i}\varvec{d}\varvec{e}\varvec{n}\varvec{t}}=\:$Wavevector component of incident light along the interface. At a flat metal-dielectric interface, the SPP wavevector is larger than that of free-space light^33^.

2 $$\:{\varvec{k}}_{\varvec{S}\varvec{P}\varvec{P}}={\varvec{k}}_{0}\sqrt{\frac{{\varvec{\epsilon}}_{\varvec{m}}{\varvec{\epsilon}}_{\varvec{d}}}{{\varvec{\epsilon}}_{\varvec{m}}+{\varvec{\epsilon}}_{\varvec{d}}}}$$

Where $\:{\varvec{\epsilon}}_{\varvec{m}}=$ permittivity of the metal. $\:{\varvec{\epsilon}}_{\varvec{d}}=$ permittivity of the dielectric. Direct excitation of SPPs by free-space light is impossible without compensating for the momentum mismatch. Nanoparticles scatter light with extra momentum, enabling localised SP excitation.

The reasons for the TE mode’s superior broadband performance are that the permittivity ($\:{\varvec{\epsilon\:}}_{\varvec{r}}$) and permeability ($\:{\varvec{\mu\:}}_{\varvec{r}}$) of the constituent materials, along with the specific geometry of the device, determine how TE and TM waves propagate and the interaction with material properties and geometry. While TM modes are typically associated with SPPs at metal-dielectric interfaces, TE modes can excite other types of plasmonic or guided modes, particularly in the proposed structure in SPR, which have broader spectral responses.

**Fig. 2:**
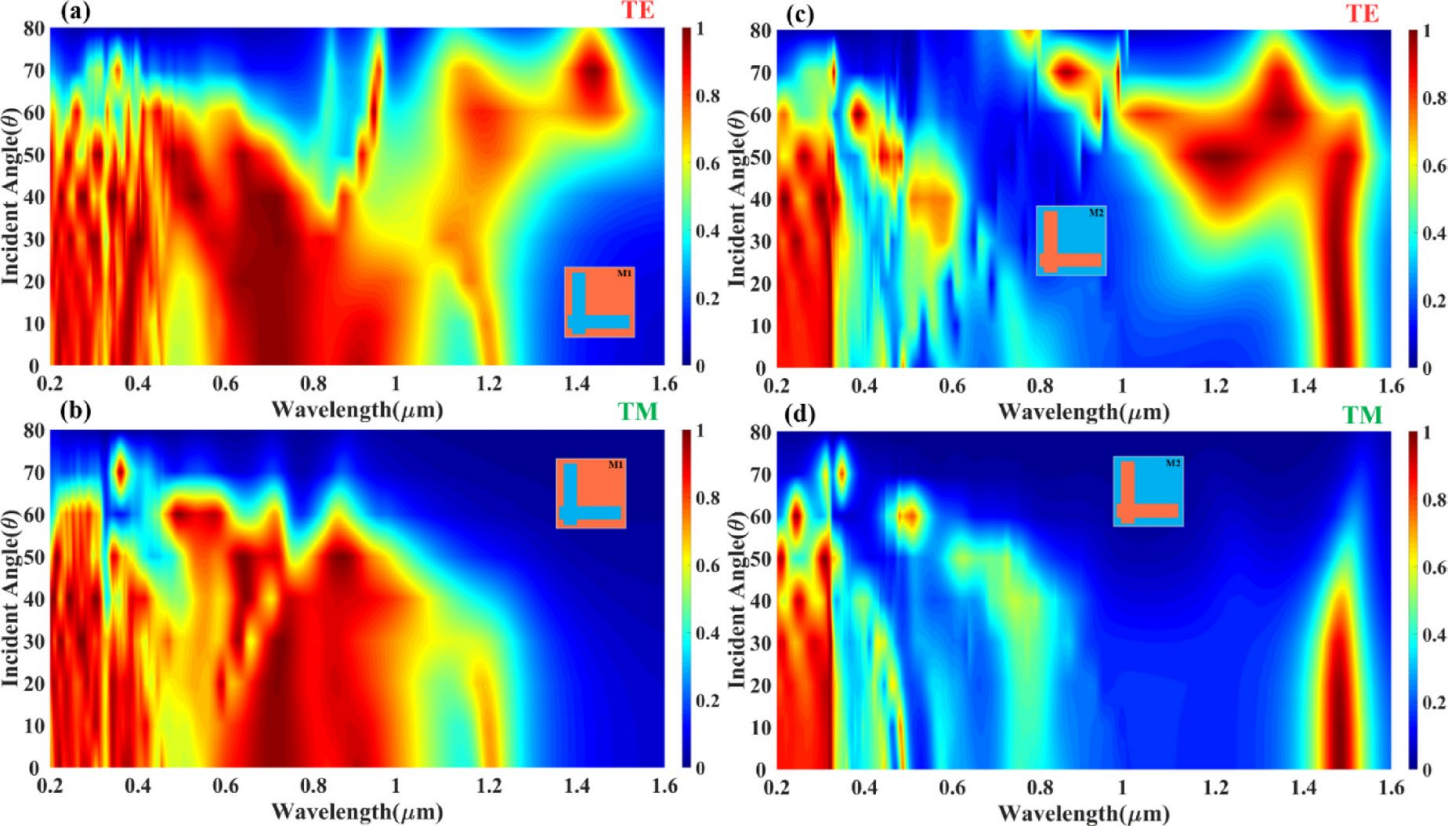
Variation in the absorption for the different modes of the input incident wave (**a**) TE and (**b**) TM for the Mode M2 (**c**) TE and (**d**) TM for the Mode M2 resonator-based absorber structure.

Figure 2 provides a detailed numerical analysis of the absorption performance of the Design M1 (L-shaped resonator) and Design M2 (complementary L-shaped resonator) absorber under different incident wave polarisation modes, TE (Transverse Electric) and TM (Transverse Magnetic) modes. The study investigates the absorption behaviour as a function of wavelength (0.2 μm to 1.6 μm) and incident angle (0° to 80°), using Ag (silver) as the resonator material. The heatmaps from Fig. 2(a, b) indicate that the absorber exhibits high absorption stability up to 60° of incident angle for wavelengths below 1.0 μm, making it highly efficient for practical applications where wide-angle light absorption is required. In Fig. 2(a) (TE mode), strong absorption (represented by the red regions) is observed in the wavelength range of 0.3 μm to 1.0 μm, with an absorption amplitude above 80% for angles up to 60°. The absorption decreases gradually beyond 60°, particularly for wavelengths above 1.2 μm, where the reflection and transmission losses become dominant. It suggests that the TE polarisation mode supports broadband absorption at lower angles, making it ideal for applications requiring efficient energy capture across a wide spectral range. The structured resonance pattern seen in the heatmap confirms that the L-shaped resonator efficiently couples with the incident wave, leading to sustained absorption performance. In Fig. 2(b) (TM mode), the absorption characteristics follow a similar trend but show slightly reduced efficiency at higher angles compared to the TE mode. The heatmap indicates that for wavelengths below 1.0 μm, the absorption remains above 80% for angles up to 50°, with minor attenuation occurring beyond this angle. The absorption spectrum for TM mode also extends towards longer wavelengths (up to 1.2 μm), suggesting that the resonator supports enhanced field confinement for TM polarisation at lower angles. The shift in absorption behaviour for TM mode, particularly at larger angles, highlights the impact of polarisation-dependent resonance conditions, where surface plasmon excitations play a significant role in modulating absorption efficiency.

The Fig. 2(c, d) presents the numerical analysis of the absorption characteristics of Design M2 (complementary L-shaped resonator) under different incident wave polarisation modes, TE (Transverse Electric) mode (Fig. 2(c)) and TM (Transverse Magnetic) mode (Fig. 2(d)). The analysis is conducted for an incident angle range of 0° to 80° over a wavelength spectrum of 0.2 μm to 1.6 μm, with Ag (silver) as the resonator material. The heatmaps illustrate the variation in absorption intensity for both incident angle and wavelength, highlighting the polarisation and angle-dependent nature of absorption in this design. The results reveal that constant high absorption is sustained up to 50° of the incident wave angle for specific wavelength bands, particularly for wavelengths < 0.4 μm and < 1.4 μm, indicating a short-band perfect absorption behaviour that is sensitive to polarisation and incidence angle.

In Fig. 2(c) (TE mode absorption response), the heatmap shows that absorption remains relatively high in the short-wavelength region (< 0.4 μm) and in a narrowband region around 1.2 μm to 1.4 μm. The absorption intensity remains above 80% for angles up to 50°, beyond which the absorption efficiency gradually decreases. The structured resonance bands observed in the heatmap suggest that TE-polarised waves interact strongly with the complementary L-shaped resonator, generating localised plasmonic excitations that contribute to the high absorption in specific spectral regions. However, for longer wavelengths (> 1.4 μm), the absorption significantly declines, confirming that Design M2 supports wavelength-selective absorption rather than broadband absorption. In Fig. 2(d) (TM mode absorption response), a similar trend is observed, but with notable differences in the distribution of absorption regions. The heatmap reveals that the short-band perfect absorption effect is prominent at shorter wavelengths (< 0.4 μm) and in the 1.0 μm to 1.4 μm range, with a consistent absorption amplitude up to 50° of the incident angle. Beyond 50°, absorption drops off significantly, particularly for wavelengths above 1.5 μm, suggesting that the resonator exhibits strong polarisation sensitivity, making it ideal for polarisation-selective optical applications. Unlike TE mode, where multiple absorption bands are present, the TM mode shows localised absorption peaks concentrated at specific resonance wavelengths, indicating different resonance coupling mechanisms for TE and TM waves. The presented design utilises a multi-layered metal-dielectric-metal (MDM) structure integrated with a topological insulator (Bi₂Sb₀.₇TeₓSeₓ) layer to achieve broadband, polarisation-insensitive optical absorption across visible to near-infrared wavelengths. The nanostructured top metal layer enables localised surface plasmon resonances, while the dielectric layer (Si/SiO₂/InP) supports Fabry–Pérot cavity modes, both enhancing light confinement and absorption within the active region. The topological insulator further contributes strong intrinsic absorption due to its surface states and narrow bandgap. Variations between Design M1 and M2 arise from geometrical tuning of the surface patterns, affecting the spectral resonance response. The observed reduction in absorption beyond ~ 60° incident angle is primarily due to inefficient coupling of incident light into the resonant modes and increased reflection—particularly under TE polarisation—highlighting angular dependence typical of such metamaterial.

**Fig. 3:**
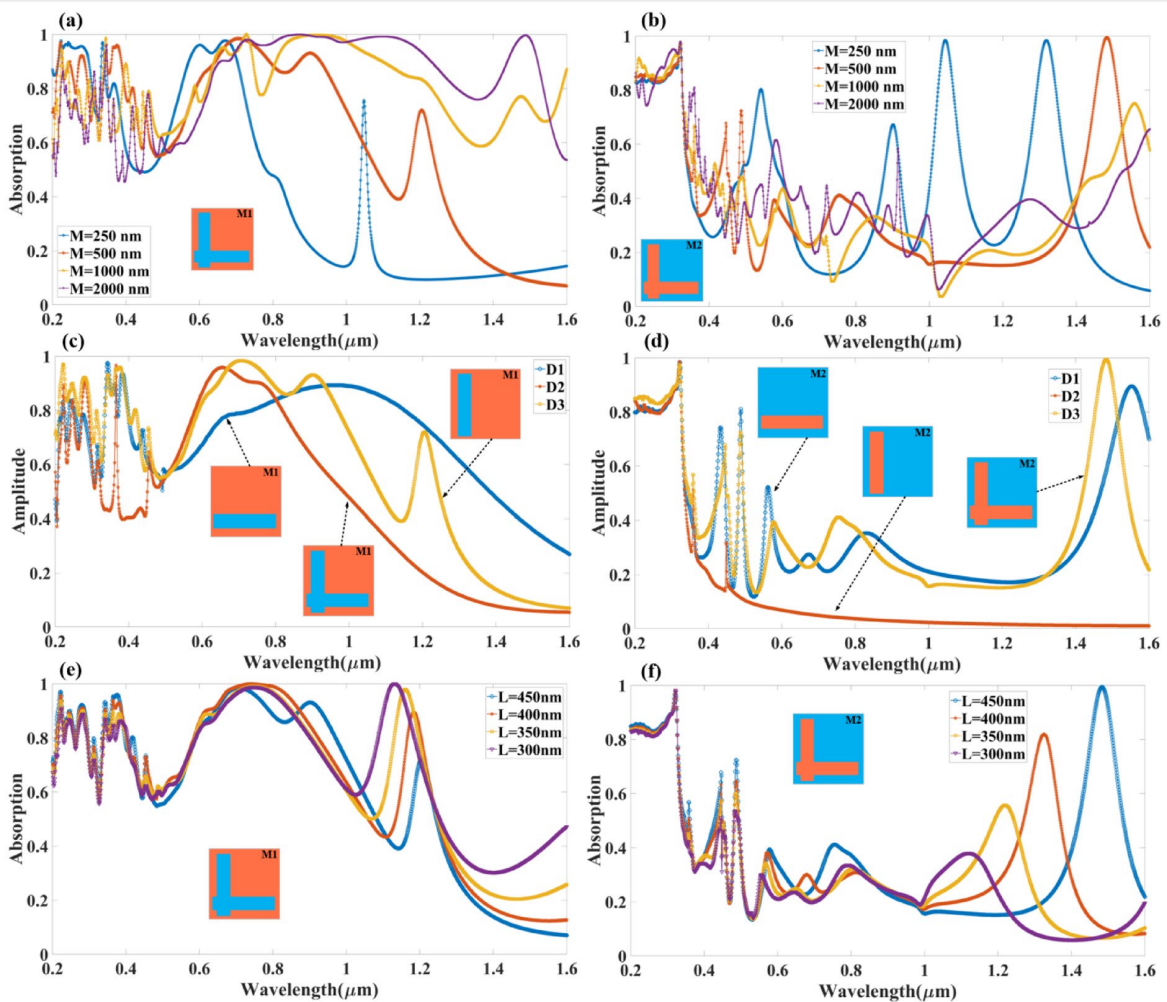
Variation in the absorption amplitude for the different resonator conditions of the proposed absorber structure. The absorption spectrum for the computational wavelength spectrum for (**a**) Design M1 and (**b**) Design M2 for the unit cell size. (**c**) Design M1 (**b**) Design M2 for single horizontal, single vertical and L-shaped modes. (**e**) Design M1 (**f**) Design M2 for the different values of the resonator length (L), changing from 300 nm to 450 nm for the computational condition with the consideration of Ag as the resonator material.

Moreover, variations in layer thickness, especially of the topological insulator and oxide, alter the effective index experienced by the confined mode. A thicker high-index TI layer increases the effective index, enhancing mode confinement and thereby red shifting and potentially narrowing the resonance due to better coupling efficiency. Conversely, a thinner dielectric reduces confinement, resulting in blue shifts and broader resonances. The metal-dielectric-metal configuration further supports localised surface plasmon resonances (LSPRs) and gap plasmon modes, which are highly sensitive to the geometric dimensions. Overall, resonance tuning through thickness control enables spectral selectivity and absorption enhancement, with each shift being a direct consequence of changes in the effective index and modal field distribution. In the parametric studies of the proposed multilayer absorber, the observed optical trends—such as resonance peak positions and bandwidth variations—can be physically explained through fundamental resonance conditions and material-optical interactions. The primary resonance condition governing the structure is the Fabry–Pérot-like interference criterion, often expressed as 2nL = mλ, where n is the effective refractive index of the dielectric layer (TI/oxide), L is the optical path length (thickness), λ is the resonant wavelength, and mm is the mode number. As the thickness of a dielectric or spacer layer increases, the optical path length increases, shifting the resonance condition to longer wavelengths (redshift) (Fig. 3). This shift occurs because a thicker dielectric layer supports lower energy (longer wavelength) modes due to increased phase accumulation. Moreover, variations in layer thickness, especially of the topological insulator and oxide, alter the effective index experienced by the confined mode. A thicker high-index TI layer increases the effective index, enhancing mode confinement and thereby redshifting and potentially narrowing the resonance due to better coupling efficiency. Conversely, a thinner dielectric reduces confinement, resulting in blue shifts and broader resonances. The metal-dielectric-metal configuration further supports localised surface plasmon resonances (LSPRs) and gap plasmon modes, which are highly sensitive to the geometric dimensions. Overall, resonance tuning through thickness control enables spectral selectivity and absorption enhancement, with each shift being a direct consequence of changes in the effective index and modal field distribution. The shift in the absorption peak is observed in the multiple parametric sweep conditions as shown in Fig. 3.

### Effect of physical parameters on absorption variation

Figure 3 presents a comprehensive numerical analysis of the absorption characteristics for different unit cell sizes, three different configurations of cell design, and the length (L) of the proposed TI-based wideband absorber. The study investigates the absorption spectrum in the computational wavelength range of 0.2 μm to 1.6 μm for two distinct resonator structures, Design M1 and Design M2. In the Fig. 3(a, b). The unit cell sizes considered for numerical evaluation range from 250 nm to 2000 nm, allowing us to observe the impact of geometric scaling on the absorption behaviour of the designed structures. The results indicate that the resonator geometry and its periodicity play a crucial role in determining the absorption characteristics, shifting resonance frequencies, and modifying the overall absorption amplitude.

In Fig. 3(a), the absorption spectrum for Design M1 (L-shaped resonator) is shown for varying unit cell sizes. It is evident from the numerical results that the L-shaped resonator contributes to a broadband absorption response, covering a significant portion of the visible and near-infrared spectrum. For a unit cell size of M = 250 nm (blue curve), the absorption remains above 80% for most of the wavelength range, with distinct peaks observed at 0.35 μm, 0.6 μm, and 1.0 μm. As the unit cell size increases to M = 500 nm (orange curve), the absorption bandwidth remains high, but the resonant peaks shift slightly towards longer wavelengths, particularly around 0.75 μm and 1.2 μm. For M = 1000 nm (yellow curve), the broadband absorption effect continues but with more pronounced resonances forming near 0.9 μm and 1.4 μm, suggesting that increasing unit cell size enhances interaction at higher wavelengths. When the unit cell size reaches M = 2000 nm (purple curve), the absorption characteristics show a further redshift, with new peaks forming at 1.3 μm and 1.5 μm, while absorption in the shorter wavelength region (below 0.4 μm) slightly decreases. These results confirm that the L-shaped resonator structure is highly effective in sustaining broadband absorption, which can be tuned further by adjusting the periodicity of the unit cell. The structure exhibits strong electromagnetic wave confinement, leading to a high absorption rate across a wide range of wavelengths. In contrast, Fig. 3(b) presents the absorption characteristics for Design M2 (complementary L-shaped resonator), which exhibits a distinctly different absorption response compared to Design M1. Unlike the broad absorption observed in Design M1, Design M2 generates multiple sharp absorption peaks, indicative of its ability to absorb electromagnetic waves at discrete resonant wavelengths selectively. For M = 250 nm (blue curve), the absorption remains relatively high in the shorter wavelength range (0.2 μm to 0.6 μm), but the response becomes oscillatory at longer wavelengths, forming several sharp resonances near 0.75 μm, 1.0 μm, and 1.4 μm. As the unit cell size increases to M = 500 nm (orange curve), the absorption peaks become more spaced out, with dominant peaks occurring near 0.8 μm, 1.1 μm, and 1.5 μm. When the unit cell reaches M = 1000 nm (yellow curve), the spectral distribution of the peaks shifts further towards longer wavelengths, with prominent absorption at 0.9 μm, 1.2 μm, and 1.6 μm. Finally, for M = 2000 nm (purple curve), the complementary L-shaped resonator generates well-defined, sharp absorption peaks at longer wavelengths, including 1.0 μm, 1.3 μm, and 1.5 μm, while absorption in the shorter wavelength region remains relatively low. This behaviour highlights that Design M2 is more sensitive to resonant coupling effects and supports multiple short-band absorption peaks instead of providing continuous broadband absorption like Design M1.

From a comparative perspective, Design M1 proves to be more effective for applications requiring continuous wideband absorption, making it highly suitable for applications such as solar energy harvesting, thermal emitters, and infrared detection, where absorption across a broad wavelength range is crucial. On the other hand, Design M2, with its multiple narrowband absorption peaks, is better suited for applications like spectral filtering, sensing, and wavelength-selective photodetectors, where specific wavelengths need to be selectively absorbed while allowing others to pass through. The impact of unit cell scaling in both designs is evident, as increasing the unit cell size leads to red shifting of absorption peaks and influences the resonance conditions by altering the effective periodicity and the interaction between neighbouring unit cells.

The Fig. 3(c, d) presents an in-depth numerical analysis of the impact of different resonator configurations—specifically, single horizontal, single vertical, and L-shaped resonators—on the absorption spectrum of the proposed wideband absorber. This study explores two absorbent designs, namely Design M1 and Design M2. Design M1 features an L-shaped resonator, and Design M2 features a complementary L-shaped resonator. The absorption spectrum is analysed across a 0.2 μm to 1.6 μm wavelength range, considering three distinct resonator configurations (D1, D2, and D3).

Figure 3(c) shows the effect of the variation in resonator geometry of Design M1 (L-shaped resonator). The D1 resonator-based structure maintains a broadband absorption profile with a minimum value above 0.7 across a wide wavelength range (0.3 μm to 1.2 μm). However, absorption weakens beyond 1.3 μm, indicating reduced electromagnetic wave confinement at longer wavelengths. The D2 resonator-based curve shows the absorption peaks at 0.5 μm, 0.9 μm, and 1.4 μm. It suggests that vertical structuring enhances wavelength selectivity while still providing moderate broadband absorption. D3 resonator (yellow curve) features an L-shaped structure, significantly improving broadband absorption and shifting peaks toward longer wavelengths. It achieves absorption above 0.8 over a broad range (0.3 μm to 1.5 μm), with distinct peaks at 0.8 μm, 1.1 μm, and 1.5 μm. The L-shape introduces additional resonance pathways, broadening the absorption bandwidth. It makes Design M1 with the D3 resonator an optimal choice for broadband applications such as solar energy harvesting, thermal emitters, and wideband photo detectors.

Figure 3(d) presents the absorption characteristics of Design M2 (complementary L-shaped resonator), which exhibits a more discrete absorption response with multiple narrowband absorption peaks rather than a continuous broadband spectrum. D1 resonator (blue curve) based design shows a simple horizontal aperture that shows high absorption in the shorter wavelength range (0.2 μm to 0.6 μm), but its absorption amplitude drops significantly beyond 0.8 μm. It suggests that horizontal apertures mainly support short-wavelength resonances but are less effective for longer wavelengths. D2 resonator (red curve) based design shows a vertical aperture, which results in a more uniform absorption response across the spectrum, with absorption peaks at 0.4 μm, 0.7 μm, and 1.3 μm. This configuration enhances resonance coupling across multiple spectral regions. The L-shaped aperture (D3 resonator (yellow curve)) introduces multiple short-band absorption peaks across the wavelength range, with strong absorption at 0.5 μm, 0.9 μm, 1.2 μm, and 1.6 μm. It demonstrates the ability of the complementary L-shaped resonator to support multiple resonance modes.

The Fig. 3(e, f) presents a detailed numerical investigation into the effect of resonator length (L) on the absorption characteristics of the proposed wideband absorber for both Design M1 (L-shaped resonator) and Design M2 (complementary L-shaped resonator) over the computational wavelength range of 0.2 μm to 1.6 μm. The resonator length L is varied from 300 nm to 450 nm, with Ag (silver) used as the resonator material, while maintaining a consistent bottom resonator and dielectric layer configuration. The resonator length plays a crucial role in modulating the resonance frequency, shifting absorption peaks, and optimising broadband absorption, making it an essential parameter for fine-tuning the absorber’s optical response. The objective of this study is to observe how variations in L impact broadband absorption in Design M1 and wavelength-selective absorption in Design M2, providing insights for applications such as energy harvesting, optical filtering, and infrared detection.

In Fig. 3(e) (Design M1 – L-shaped resonator), the absorption spectrum demonstrates strong broadband behaviour, with variations in L primarily shifting absorption peaks across the wavelength range. The L = 450 nm (blue curve) exhibits high absorption (> 80%) across a broad spectrum, particularly between 0.3 μm and 1.2 μm, with dominant peaks forming at 0.75 μm, 1.1 μm, and 1.4 μm. As the resonator length is reduced to L = 400 nm (orange curve), the broadband response remains stable, but with a noticeable shift in peak positions, particularly in the 0.8 μm to 1.3 μm range, confirming that reducing L modifies the resonance conditions. With L = 350 nm (yellow curve), the absorption remains high, but distinct peaks emerge at 0.9 μm and 1.2 μm, suggesting that further reductions in resonator length introduce additional resonance modes. The L = 300 nm (purple curve) configuration shows a more redshifted response, with peaks forming at 1.0 μm and 1.5 μm, indicating that a shorter resonator length enhances absorption at longer wavelengths. These results confirm that increasing L enhances broadband absorption at shorter wavelengths, while decreasing L shifts the resonance peaks towards longer wavelengths, making L an effective parameter for tuning the operational spectrum. The strong broadband response in Design M1 suggests its suitability for applications such as solar energy harvesting, photodetection, and thermal emission.

In Fig. 3(f) (Design M2 – Complementary L-shaped resonator), the absorption spectrum exhibits multiple distinct absorption peaks, with variations in L strongly influencing the positioning and intensity of resonances. For L = 450 nm (blue curve), strong absorption is observed at 0.4 μm, 0.9 μm, and 1.3 μm, confirming the presence of well-defined resonance conditions. As L decreases to 400 nm (orange curve), the peaks shift slightly, with dominant absorption occurring at 1.0 μm and 1.4 μm, highlighting the wavelength-selective nature of the resonator. With L = 350 nm (yellow curve), additional peaks emerge, particularly at 0.8 μm, 1.2 μm, and 1.5 μm, confirming that reducing L introduces new resonance modes that enhance multi-wavelength selectivity. The L = 300 nm (purple curve) configuration exhibits the most pronounced redshift, with peaks at 0.9 μm, 1.3 μm, and 1.6 μm, indicating that shorter resonator lengths favour longer-wavelength resonance coupling. Unlike Design M1, which maintains broadband absorption, Design M2 exhibits sharp, well-defined absorption peaks, making it ideal for narrowband optical filtering, biosensing, and wavelength-selective detection.

**Fig. 4:**
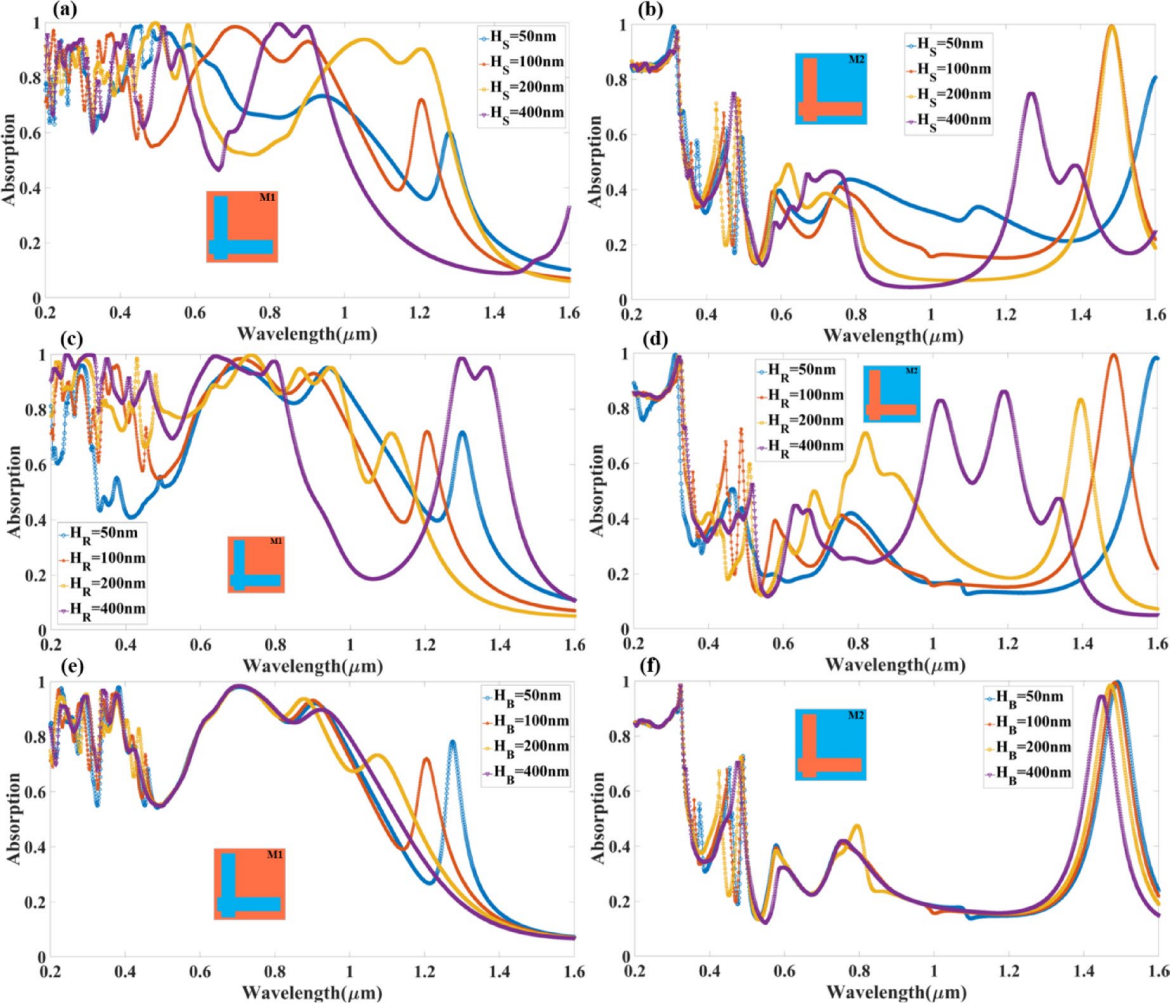
Variation in the amplitude of the absorption for the different values of the (**a**,**b**) Silicon dioxide height (H_s_). (**c**,**d**) the top resonator layer (H_R_). (**e**,**f**) the bottom resonator layer (H_B_) for both types of resonator modes (M1, M2).

Figure 4 presents the effect of resonance conditions and overall absorption performance by adjusting the height of the dielectric spacer thickness (H_s_), the height of the resonator (H_R_) and the bottom resonator layer thickness (H_B_) across the 0.2 to 1.6 μm wavelength range. This study offers a technique to optimise photonic devices for use such as solar energy harvesting, infrared detection, and optical filtering by revealing how to tune the absorber’s effectiveness by varying the thickness of the dielectric layer.

From the Fig. 4(a), we can see the Design M1, which features an L-shaped resonator, the absorption spectrum exhibits strong broadband characteristics that shift with varying H_s_ values between 50 nm, 100 nm, 200 nm, and 400 nm. The blue curve (H_s_ = 50 nm) shows a relatively uniform absorption above 80% across a wide wavelength range, particularly between 0.3 μm and 1.2 μm, but with slightly weaker absorption beyond 1.3 μm. As the dioxide height increases to H_s_ = 100 nm (red curve), the absorption spectrum remains broad but introduces new resonances around 0.9 μm and 1.4 μm, indicating improved coupling of incident electromagnetic waves to the plasmonic resonator. With H_s_ = 200 nm (yellow curve), additional distinct absorption peaks emerge at 0.75 μm, 1.1 μm, and 1.5 μm, suggesting that an optimal H_s_ enhances the absorber’s efficiency by strengthening the resonance conditions. The most significant effect is observed when H_s_ = 400 nm (purple curve), where strong absorption peaks appear at longer wavelengths, particularly at 1.3 μm and 1.5 μm, while some absorption in the shorter wavelength range decreases slightly. This trend suggests that increasing H_s_ shifts resonance conditions towards longer wavelengths, as a thicker dioxide layer modifies the effective optical path length and changes the phase-matching conditions between the resonator and dielectric layers.

From the Fig. 4(b), we can see Design M2, which features a complementary L-shaped resonator, the absorption response exhibits a distinctly different pattern, with multiple sharp absorption peaks instead of a continuous broadband spectrum. The blue curve (H_s_ = 50 nm) shows a relatively high absorption in the short-wavelength range (0.2 μm to 0.6 μm), while the absorption beyond 0.8 μm is oscillatory, suggesting that a very thin dioxide layer supports resonance primarily at shorter wavelengths. As H_s_ increases to 100 nm (red curve), new absorption peaks emerge at 0.8 μm and 1.2 μm, highlighting the shift in resonance conditions. With H_s_ = 200 nm (yellow curve), the spectral distribution of the absorption peaks becomes more distinct, with strong peaks at 0.5 μm, 1.0 μm, and 1.4 μm, indicating enhanced coupling between the resonator aperture and the dielectric interface. For the thickest dioxide layer (H_s_ = 400 nm, purple curve), the absorption spectrum exhibits well-defined peaks at 0.9 μm, 1.3 μm, and 1.5 μm, confirming that a thicker spacer layer shifts the resonance modes towards longer wavelengths while modifying their intensity. Unlike Design M1, where a thicker dielectric leads to broadband absorption, Design M2 primarily generates multiple, discrete resonance peaks, making it ideal for narrowband optical filtering applications. Figure 4(c, d) illustrates a study that considers H_R_ values ranging from 50 nm to 400 nm, using Ag (silver) as the material for the top resonator, while the dioxide layer and bottom resonator are composed of Si (silicon) and Ag, respectively.

In Fig. 4(c) (Design M1), increasing H_R_ results in enhanced broadband absorption, with the 400 nm layer (purple curve) achieving the highest absorption across a wide wavelength range. The absorption spectrum exhibits strong peaks at 0.75 μm, 1.0 μm, and 1.4 μm, with increased resonance shifting towards longer wavelengths as the resonator thickness increases.

In Fig. 4(b) (Design M2), the complementary L-shaped resonator exhibits multiple sharp absorption peaks, with H_R_ = 400 nm generating dominant absorption at 1.0 μm, 1.3 μm, and 1.5 μm, confirming the strong wavelength-selective behaviour of this structure.

One of the most important design parameters for maximising both broadband and wavelength-selective absorption is resonator length (L), which can be adjusted to fine-tune the absorption spectrum. The two resonator structures exhibit different absorption tendencies when L is consistently varied from 300 nm to 450 nm in the numerical study. Absorption efficiency is further increased by strengthening resonance coupling through an increase in H_R_. This tunability makes it possible to tailor metamaterial-based photonic devices for particular wavelength ranges by precisely constructing optical responses.

Figure 4(e, f) presents a numerical analysis of how varying the bottom resonator layer thickness (H_B_) influences the absorption characteristics in both Design M1 and Design M2 across the 0.2 to 1.6 μm wavelength range. The H_B_ values are varied between 50 nm, 100 nm, 200 nm, and 400 nm with Ag (silver) as the material for the bottom resonator. The bottom resonator layer plays a crucial role in controlling the reflectivity and absorption efficiency by enhancing the coupling of incident light into the resonator structure, leading to optimised resonance conditions.

In Fig. 4(e) (Design M1 – L-shaped resonator), the absorption spectrum exhibits relatively stable broadband behaviour across all values of H_B_, confirming that the bottom resonator thickness does not drastically alter the broadband absorption response. For H_B_ = 50 nm (blue curve), the absorption remains high (> 80%) in the shorter wavelength range (0.3 μm to 1.2 μm) but slightly decreases beyond 1.3 μm. As the bottom resonator thickness increases to H_B_ = 100 nm (red curve), there is an improvement in absorption across the entire spectrum, with well-defined peaks forming around 0.75 μm, 1.1 μm, and 1.4 μm, indicating enhanced resonance coupling. For H_B_ = 200 nm (yellow curve), a further shift in absorption peaks is observed, with stronger absorption in the 1.0 μm to 1.5 μm region, suggesting that increased bottom resonator thickness enhances absorption at longer wavelengths by modifying the plasmonic resonance conditions. The H_B_ = 400 nm (purple curve) scenario exhibits similar absorption behaviour, with further redshifted resonances around 1.2 μm and 1.5 μm, confirming that a thicker bottom resonator enhances light confinement at higher wavelengths while maintaining broadband absorption. The results suggest that for broadband applications, an optimal range of H_B_ between 100 nm and 200 nm provides a balanced trade-off between absorption intensity and spectral coverage, making Design M1 highly efficient for solar energy harvesting, photothermal conversion, and infrared sensing applications.

In Fig. 4(f) (Design M2 – Complementary L-shaped resonator), the absorption spectrum exhibits multiple sharp resonances instead of broadband behaviour, indicating strong wavelength-selective absorption. For H_B_ = 50 nm (blue curve), the absorption is relatively uniform in the shorter wavelength range (0.2 μm to 0.6 μm) but shows multiple absorption peaks beyond 0.8 μm, with dominant peaks appearing at 1.0 μm and 1.4 μm. Increasing the bottom resonator thickness to H_B_ = 100 nm (red curve) leads to the formation of additional absorption peaks, particularly at 0.9 μm, 1.2 μm, and 1.5 μm, confirming improved resonance coupling. With H_B_ = 200 nm (yellow curve), the spectrum becomes even more structured, with pronounced peaks at 0.75 μm, 1.0 μm, 1.3 μm, and 1.5 μm, suggesting that intermediate thickness values enhance multi-wavelength absorption. The H_B_ = 400 nm (purple curve) scenario exhibits further refinement in absorption peaks, confirming the ability of a thicker bottom resonator layer to tune resonance frequencies while maintaining strong selective absorption at 1.0 μm, 1.3 μm, and 1.5 μm. Unlike Design M1, which favours broadband absorption, Design M2 exhibits sharp and tunable resonance peaks, making it highly suitable for narrowband filtering, biosensing, and wavelength-selective photodetection applications.

**Fig. 5:**
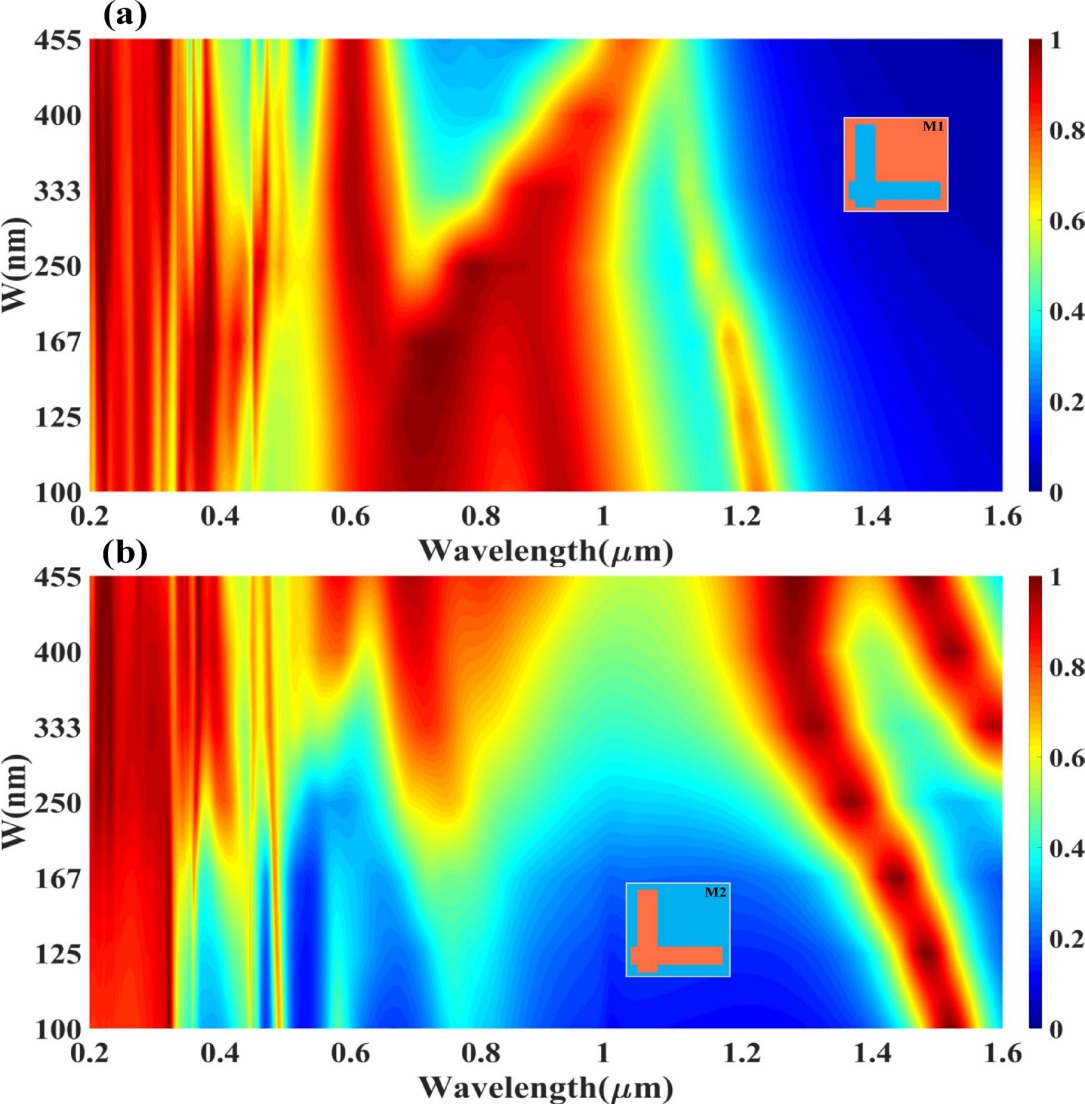
Comparative study of the absorption spectrum for the (**a**) Design M1 and (**b**) Design M2 of the resonator structure for the different values of the resonator width (W).

Figure 5 presents a comparative numerical study of the absorption spectrum for Design M1 (L-shaped resonator) and Design M2 (complementary L-shaped resonator) with varying resonator width (W) over the wavelength range of 0.2 μm to 1.6 μm. The resonator width (W) is varied from 100 nm to 455 nm, using Ag (silver) as the resonator material, to analyse its effect on the absorption performance. The absorption heatmaps clearly illustrate that increasing the resonator width results in a shift in the resonance wavelength, significantly impacting absorption intensity across different spectral regions.

In Fig. 5(a) (Design M1), strong absorption is observed across shorter wavelengths (0.2 μm to 0.8 μm), while for W > 250 nm, absorption extends to longer wavelengths (> 0.7 μm), with a noticeable resonance shift. The absorption efficiency remains high, particularly in the visible and near-infrared range, demonstrating broadband absorption behaviour.

In contrast, Fig. 5(b) (Design M2) exhibits a distinct shift in resonance, where higher absorption occurs for wavelengths > 1.0 μm as W increases, confirming the strong dependency of wavelength-selective absorption on the resonator width. The difference in absorption characteristics between Design M1 and M2 suggests that Design M1 favours broadband absorption, while Design M2 supports narrowband, tunable resonance peaks. This width-dependent tuning capability is essential for designing adaptive photonic devices, such as infrared sensors, multi-wavelength filters, and energy-harvesting structures, where precise spectral control is required. The observed resonance shift for increasing W provides a powerful approach for customising optical absorption properties, making these resonator structures highly versatile for metamaterial-based photonic applications.

**Fig. 6:**
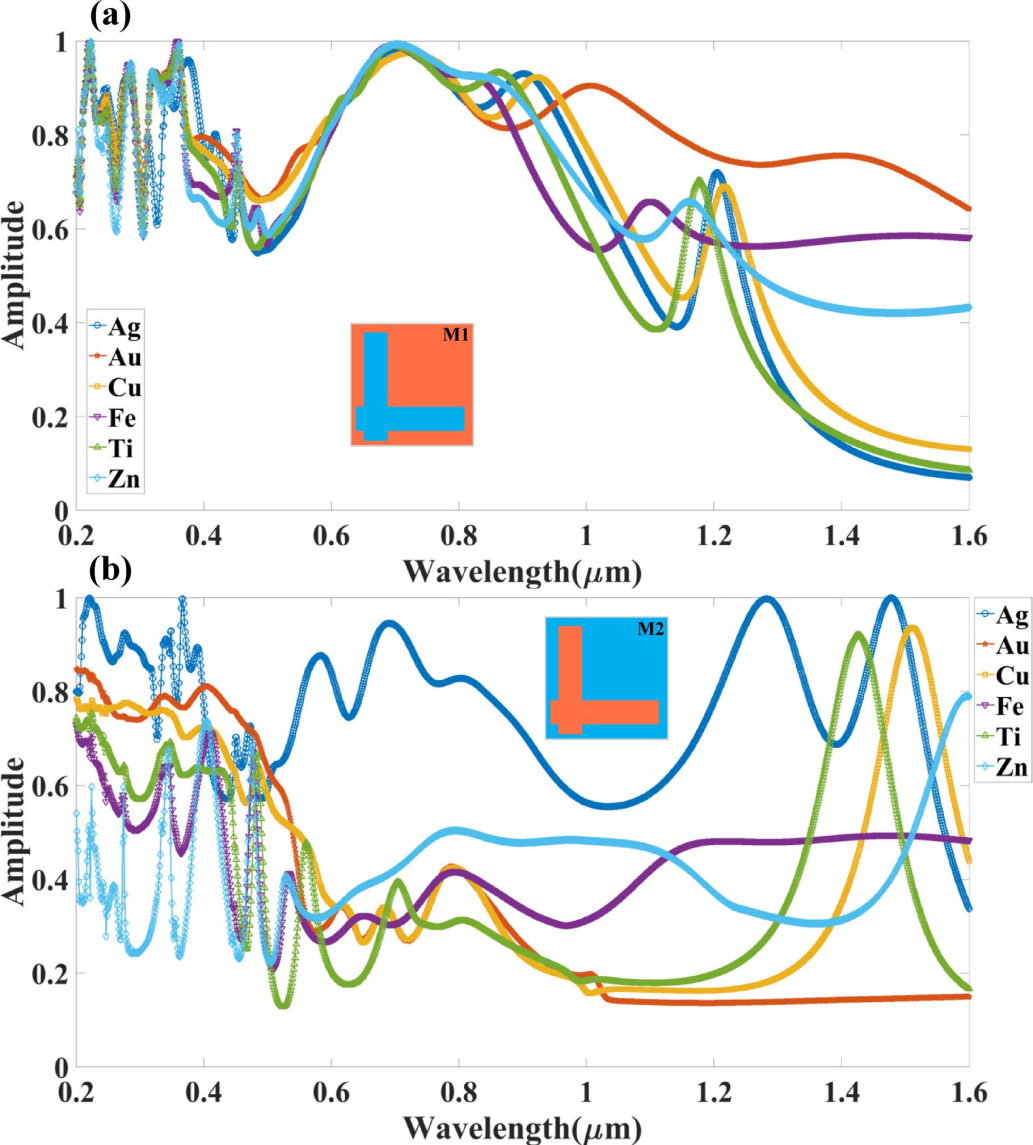
Variation in the absorption for the different metals considered as top (H_R_) and bottom (H_B_) layers for (**a**) Mode M1 and (**b**) Mode M2 resonator-based absorber structure. It is observed that in the Mode M1 design of the structure, the wide band absorber is observed for the Au material. The multiband with an amplitude of > 50% is observed for the Ag material in Mode M2 of the resonator design.

The Fig. 6, presents a numerical analysis of the effect of different metallic materials used for the top resonator layer (H_R_) and bottom layer (H_B_) on the absorption characteristics of Design M1 (L-shaped resonator) and Design M2 (complementary L-shaped resonator) over the wavelength range of 0.2 μm to 1.6 μm. The study considers six different metals—Ag (silver), Au (gold), Cu (copper), Fe (iron), Ti (titanium), and Zn (zinc)—to evaluate their impact on absorption performance. The selection of metals significantly influences the optical response, as different metals exhibit distinct plasmonic properties, affecting resonance conditions, energy confinement, and absorption bandwidth.

In Fig. 6(a) (Design M1 – L-shaped resonator), it is observed that gold (Au) exhibits the best broadband absorption performance, maintaining an absorption amplitude above 80% across a wide spectral range from 0.3 μm to 1.2 μm. This superior performance of Au is attributed to its low optical losses and strong plasmonic properties, which enhance the resonator’s ability to efficiently couple incident light. The absorption spectrum for Ag (silver) also demonstrates strong resonance peaks, particularly around 0.75 μm, 1.1 μm, and 1.4 μm, suggesting that Ag supports multiple distinct resonant modes. However, the absorption efficiency is slightly lower than that of Au at longer wavelengths. Copper (Cu) follows a similar trend but with slightly reduced absorption in the visible range due to its higher inherent optical losses. For Fe (iron), Ti (titanium), and Zn (zinc), the absorption spectrum exhibits significant variation, with Zn showing lower absorption across most of the spectrum. Ti and Fe, on the other hand, display moderate broadband absorption, particularly between 0.4 μm and 1.0 μm, confirming that they support plasmonic resonance but with increased energy dissipation losses. Overall, Design M1 with Au provides the most effective broadband absorption, making it ideal for applications such as solar energy harvesting, thermal emitters, and photodetectors.

In Fig. 6(b) (Design M2 – Complementary L-shaped resonator), the absorption behaviour is significantly different due to the structural variation in the resonator design. Unlike Design M1, which supports broadband absorption, Design M2 exhibits multiple sharp resonance peaks, indicating wavelength-selective absorption. The results show that silver (Ag) exhibits the highest absorption efficiency, maintaining an amplitude of above 50% across multiple spectral bands, particularly around 0.4 μm, 0.9 μm, 1.2 μm, and 1.5 μm. It confirms that Ag facilitates strong localised plasmonic resonances, making it suitable for applications requiring multi-wavelength filtering, sensing, and selective photo detection. Gold (Au) also performs well, but its absorption is more concentrated in the visible range (< 1.0 μm), with reduced absorption at longer wavelengths compared to Ag. Copper (Cu) follows a similar trend to Au, while Fe, Ti, and Zn exhibit more scattered absorption peaks, with lower efficiency in the near-infrared region (> 1.0 μm). The results confirm that Design M2 is more sensitive to material selection, and using Ag leads to optimised multiband absorption, making it suitable for infrared sensing, optical filtering, and wavelength-selective detection applications. The choice of metal layer—Fe, Ti, Cu, Zn, Ag, or Au—critically influences the plasmonic behaviour of the absorber structure due to their distinct optical and electronic properties. Ag and Au are widely regarded as the most efficient plasmonic materials in the visible to near-infrared range because of their low optical losses and high negative real permittivity, enabling strong surface plasmon resonance (SPR) and enhanced light confinement. Cu exhibits similar plasmonic behaviour to Au but with higher losses and oxidation susceptibility. Zn and Ti, while less conventional, can support plasmonic effects in the UV-visible range; however, their higher losses limit efficiency. Fe, being magnetic, introduces magneto-optic effects but suffers from significant damping, making it less effective for strong SPRs. The TI layer, interfaced with these metals, benefits more from Ag and Au due to better coupling efficiency and minimal interfacial losses, while metals like Ti and Fe may introduce additional damping that suppresses resonance quality. Thus, for optimal plasmonic enhancement and absorption, Ag and Au are superior choices among the listed metals.

## Impedance mode theory and metamaterial behaviour of structure

Important factors that control its electromagnetic response, especially how well it can absorb incident solar light, are the permittivity ($\:{\varvec{\epsilon\:}}_{\varvec{r}}$) and permeability ($\:{\varvec{\mu\:}}_{\varvec{r}}$). While the relative $\:{\varvec{\epsilon\:}}_{\varvec{r}}$ Indicates how the material polarises in reaction to an electric field, the relative $\:{\varvec{\mu\:}}_{\varvec{r}}$ describes how the material reacts to magnetic fields in comparison to space. By adjusting these characteristics, solar absorbers can manage the impedance matching between the absorber and the surrounding medium, reducing reflection and increasing absorption. In particular, metamaterial-based solar absorbers frequently use customised $\:{\varvec{\mu\:}}_{\varvec{r}}$ and $\:{\varvec{\epsilon\:}}_{\varvec{r}}$ values to achieve high-efficiency, wideband, and angle-insensitive absorption. An ideal match across practical permeability and permittivity is necessary for the proposed design’s absorption to enhance light–matter couplings while maintaining resonance modes to optimise energy harvesting across a wide bandwidth. We utilise the figures of S-parameters that are produced from the transmitted (S_21_) and reflected (S_11_) components from the solar absorber to calculate the effective $\:{\varvec{\mu\:}}_{\varvec{r}}$ and $\:{\varvec{\epsilon\:}}_{\varvec{r}}$ of the absorber. The link among variables and the characteristics of the material under study is depicted in Eq. 1^34^.

3 $$\:{\varvec{Z}}_{\varvec{e}\varvec{f}\varvec{f}}=\sqrt{\frac{{\left(1+{\varvec{S}}_{11}\right)}^{2}-{\varvec{S}}_{21}^{2}}{{\left(1-{\varvec{S}}_{11}\right)}^{2}-{\varvec{S}}_{21}^{2}}}=\frac{1+{\varvec{S}}_{11}}{1-{\varvec{S}}_{11}}$$

4 $$\:\varvec{n}=\frac{-\varvec{iln}\left({\varvec{e}}^{\varvec{i}\varvec{n}{\varvec{k}}_{0}\varvec{d}}\right)}{{\varvec{k}}_{\varvec{o}}\varvec{d}}$$

**Fig. 7:**
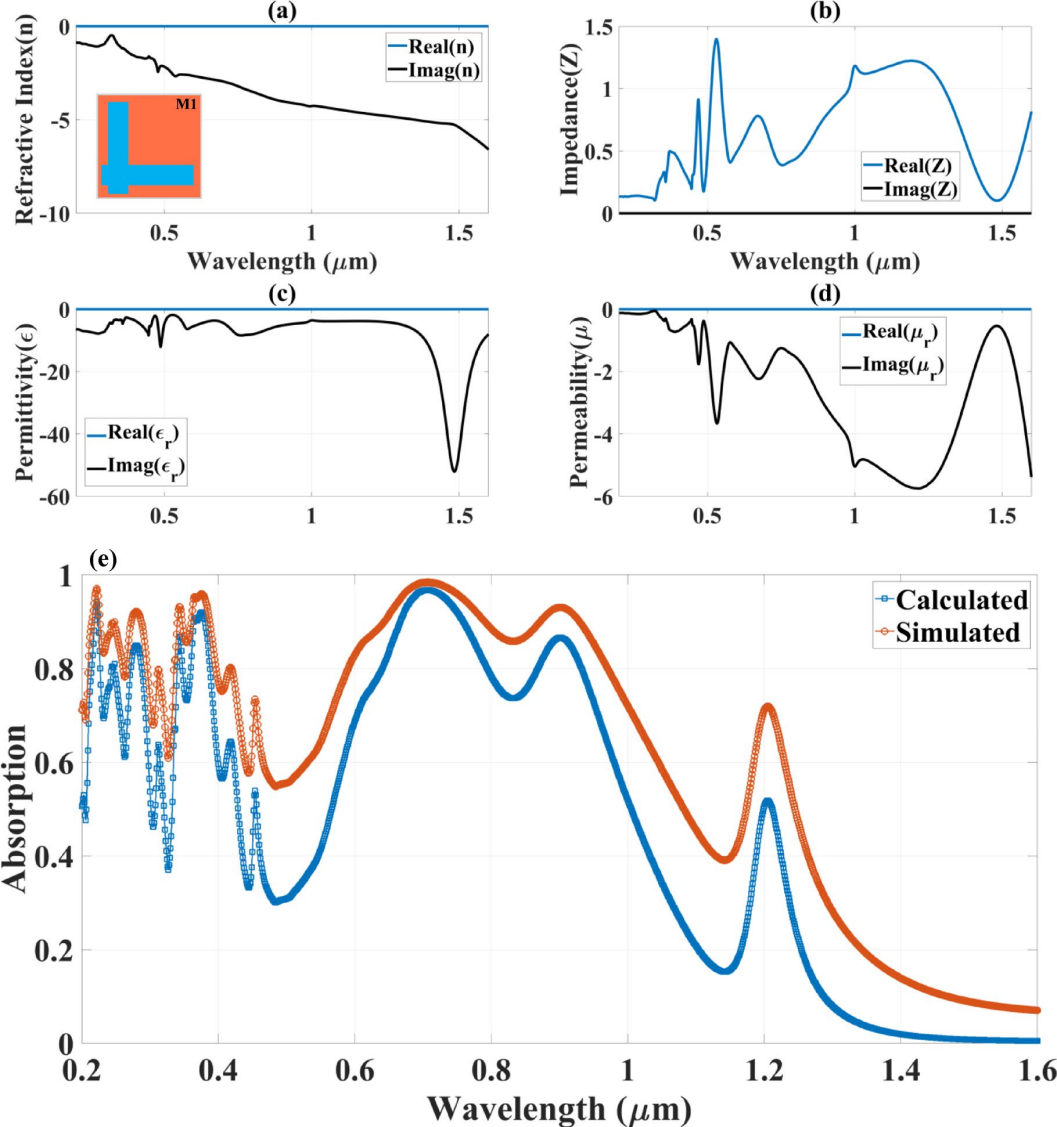
The variation in the different optical parameters (**a**) refractive index (n), (**b**) impedance (Z), (**c**) permittivity (ɛ) and (**d**) permeability (µ) for the Design M1 structure-based absorber structure.

**Fig. 8:**
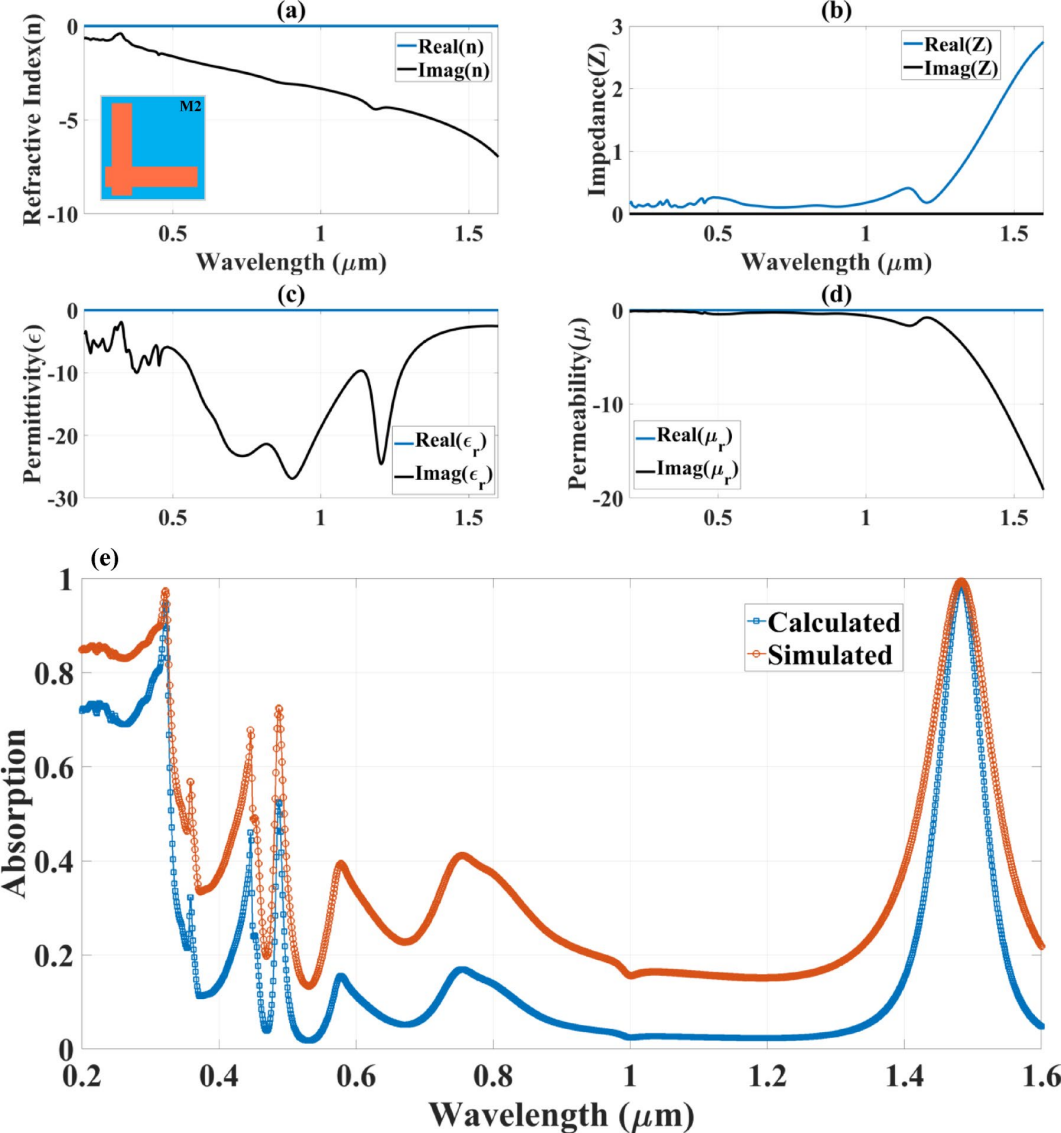
The variation in the different optical parameters (**a**) refractive index (n), (**b**) impedance (Z), (**c**) permittivity (ɛ) and (**d**) permeability (µ) for the Design M2 structure-based absorber structure.

Where $\:\varvec{d}$ is the thickness of the material$\:,\:{\varvec{k}}_{0}$​ is the free-space wavenumber, $\:{\varvec{Z}}_{\varvec{e}\varvec{f}\varvec{f}}$ is the normalised impedance of the material selected. Nevertheless, the refractive index n is determined using the given equation, as seen in Eq. 2. It is important to note that, as Eq. 3^35^ illustrates, $\:{\varvec{\mu\:}}_{\varvec{r}}$ and $\:{\varvec{\epsilon\:}}_{\varvec{r}}$ could be calculated using values of impedance and refractive index. Figures 7 and 8 show the variation in those variables for the calculated wavelength spectrum. In the formula $\:{\varvec{e}}^{\varvec{i}\varvec{n}{\varvec{k}}_{0}\varvec{d}}=\varvec{X}\pm\:\varvec{i}\sqrt{1-{\varvec{X}}^{2}}\text{}\text{where}\text{}\varvec{X}=1\:/2{\varvec{S}}_{21}\left(1-{\varvec{S}}_{11}^{2}+{\varvec{S}}_{21}^{2}\right).$

5 $$\:{\varvec{\mu\:}}_{\varvec{r}}=\varvec{n}\varvec{Z}{\varvec{\epsilon\:}}_{\varvec{r}}=\frac{\varvec{n}}{\varvec{Z}}$$

Where Z is the impedance matching.

It is crucial to take interference theory into account while figuring out the absorbance properties of multi-layered systems. The absorber can be modelled as a Fabry-Perrot interferometer, where reflections occur in the overall substrates, affecting the overall reflection that is visible. Therefore, by using Eq. 4 to solve the calculations, the overall reflection coefficient $\:\varvec{r}$ could be found^34,36^.

6 $$\:\varvec{r}={\varvec{r}}_{12}^{\varvec{{\prime\:}}}-\frac{{\varvec{t}}_{12}^{\varvec{{\prime\:}}}{\varvec{t}}_{21}^{\varvec{{\prime\:}}}{\varvec{e}}^{\varvec{i}2\varvec{\beta\:}}}{1+{\varvec{r}}_{21}^{\varvec{{\prime\:}}}{\varvec{e}}^{\varvec{i}2\varvec{\beta\:}}}$$

Where $\:{\varvec{r}}_{12}^{\varvec{{\prime\:}}}$, $\:{\varvec{t}}_{12}^{\varvec{{\prime\:}}}$, $\:{\varvec{t}}_{21}^{\varvec{{\prime\:}}}$, and $\:{\varvec{r}}_{21}^{\varvec{{\prime\:}}}\:$are the reflection and transmission coefficients of the absorber, and $\:\varvec{\beta\:}=\:\varvec{n}{\varvec{k}}_{0}\varvec{d}$ is the phase shift due to the wave propagation of the analysed material. Following this, the absorption is computed using the equation $\:\varvec{A}\left(\varvec{\lambda\:}\right)=1-\:{\left|\varvec{R}\left(\varvec{\lambda\:}\right)\right|}^{2}$. The outcomes of this formula and the simulation results are compared. Absorbance at different wavelengths is assessed using the previous formula. We measured the rate of absorption to determine the variance that could result from applying this theory with the suggested absorber. The dielectric constant, permeability, refractive index, and impedance changes across the entire wavelength range are shown in Fig. 7(a-d) and Fig. 8(a-d) for both structures, calculated from Eqs. 3–5. The variations in absorbance from the interference theory and the simulated study is shown in Fig. 7(e) and Fig. 8(e) for the both structures which is calculated form the Eq. 6. It is identified that the variations of the 10–20% amplitude shift in absorbing for the interference mode theory and finite element method in COMSOL. Designing a wideband absorber in a metal-insulator-metal structure relies on several key properties. Firstly, the geometry of the top layer supports various modes and resonances, contributing to broadband absorption^37^. Additionally, using metals with high loss enhances reflectance and light trapping between the layers. The incorporation of such metals facilitates wideband impedance matching with free space, leading to wideband absorption. The combination of metals (Fe/Ti/Cu/Zn/Ag/Au) provides enhanced plasmonic resonance and improved absorption bandwidth. The chosen insulators (Si/SiO₂/InP) as a dielectric spacer are positioned with a thickness (**H**_**s**_) above the TI material layer. offer optimal refractive index contrast and thermal stability, an essential condition for maximising absorption by minimising reflection. Impedance matching must be achieved, and the spacing is crucial to that process. The TI Bi₁.₅Sb₀.₅Te₁.₈Se₁.₂ exhibits unique surface states contributing to wideband absorption and tunability. Because of its great carrier velocity and distinct surface states, the TI substance has remarkable optical characteristics. This substance can produce high absorption effectiveness by improving light-matter coupling and reducing loss of energy^38^.

The Fig. 7, provides an in-depth analysis of the optical parameters for the Design M1 (L-shaped resonator) absorber structure, confirming its behavior as a metamaterial through the evaluation of its refractive index (n), impedance (Z), permittivity (ɛ), and permeability (µ) over a wavelength range of 0.2 μm to 1.6 μm. The investigation highlights the key properties of the absorber and its response to incident electromagnetic waves, demonstrating the presence of negative index characteristics and the magnetic-negative (MNG) mode of metamaterials. Additionally, a comparative analysis between the numerically simulated and analytically calculated absorption spectra provides validation for the absorber’s efficiency and accuracy of the computational model. MNG and DNG materials are artificial media where both effective ɛ and µ are negative over a specific frequency range. Exhibit unique electromagnetic responses. For perfect transmission (or perfect absorption in the case of a lossy material), the impedance of the metamaterial needs to be matched to the impedance of the surrounding medium (usually free space).

The impedance of free space ($\:{\varvec{Z}}_{0}$) is given by :

7 $$\:{\varvec{Z}}_{0}=\sqrt{\frac{{\varvec{\mu\:}}_{0}}{{\varvec{\epsilon\:}}_{0}}}\approx\:377\:\varvec{\varOmega\:}$$

For impedance matching to free space, the effective impedance of the metamaterial should be close to that of free space:

$$\:{\varvec{Z}}_{0}\approx\:{\varvec{Z}}_{\varvec{e}\varvec{f}\varvec{f}}$$

When $\:{\varvec{Z}}_{\varvec{e}\varvec{f}\varvec{f}}$ matches $\:{\varvec{Z}}_{0}$​, the reflection approaches zero, and the transmission is eliminated. Impedance matching is essential to minimise reflection and allow efficient coupling of incident energy into the absorber. Meanwhile, DNG and MNG properties further strengthen this effect by enabling strong field localisation.

In Fig. 7(a), the refractive index n is plotted as a function of wavelength, with both real and imaginary components shown. The results indicate that the real part of the refractive index exhibits negative values across a significant portion of the spectrum, particularly in the near-infrared region (> 0.8 μm). This negative refractive index confirms that the designed structure operates as a metamaterial, enabling unique electromagnetic wave manipulation properties such as negative refraction, sub-wavelength focusing, and enhanced energy confinement. The imaginary part of the refractive index remains relatively stable, confirming that the structure maintains low losses, which is essential for practical applications in optical sensing, energy harvesting, and photonic devices.

In Fig. 7(b), the impedance Z of the structure is analysed, demonstrating fluctuations in both its real and imaginary components over the wavelength spectrum. The variations in impedance indicate the strong coupling between the resonator and the surrounding medium, ensuring efficient energy absorption. The observed impedance characteristics contribute to the broadband absorption performance of the structure by facilitating impedance matching, minimising reflection, and enabling effective energy dissipation within the material layers.

In Fig. 7(c), the permittivity ɛ (dielectric response) is presented, showcasing its real and imaginary components. A significant feature in this graph is the negative values observed in the real part of permittivity (Re(ɛ)), confirming the metallic-like behaviour of the structure and its ability to support plasmonic resonances. The imaginary component remains within a moderate range, suggesting controlled losses, which is advantageous for applications that require high absorption efficiency with minimised dissipation. This negative permittivity behaviour is characteristic of materials that exhibit plasmonic resonances, enhancing absorption performance through localised field enhancement and resonance excitation.

In Fig. 7(d), the permeability µ of the structure is analysed, revealing that the real part of permeability (Re(µ)) also exhibits negative values, particularly in the mid-IR range (> 0.8 μm). It confirms that the structure supports the magnetic-negative (MNG) mode of metamaterials, meaning that it can manipulate magnetic field interactions, leading to enhanced resonance effects and field confinement. The simultaneous presence of negative permittivity and negative permeability classifies this structure as a double-negative (DNG) or MNG metamaterial, making it highly effective for wavefront engineering, absorption enhancement, and electromagnetic wave manipulation.

Finally, in Fig. 7(e), a comparative analysis between the calculated and simulated absorption spectra is presented, validating the accuracy of the computational model. The blue curve represents the theoretical (calculated) absorption, and the numerically simulated absorption—obtained using Finite Element Method (FEM) simulations—is represented by the orange curve. The two curves’ strong agreement verifies the design’s precision and the absorber’s ability to absorb incident energy over a wide wavelength range efficiently. Strong absorption peaks at 0.75 μm, 1.1 μm, and 1.4 μm are shown in the simulated absorption spectrum, and these roughly correspond to the theoretical predictions. The absorber’s capacity to generate high and consistent absorption across a broad range of optical frequencies is highlighted by this alignment, which makes it ideal for photonic and optoelectronic applications.

Figure 8 presents a detailed numerical analysis of the optical parameters of Design M2 (complementary L-shaped resonator), providing critical insight into its metamaterial properties. The study evaluates key parameters, including the refractive index (n), impedance (Z), permittivity (ε), and permeability (µ), across a wavelength range of 0.2 μm to 1.6 μm. These findings help characterise the resonator’s interaction with electromagnetic waves and its potential to exhibit negative index behaviour, a defining feature of metamaterials. Additionally, a comparison between the calculated and simulated absorption spectra further validates the computational model, reinforcing the absorber’s efficiency and reliability for advanced photonic applications.

The absorber’s refractive index (n), which includes both real and imaginary components, is displayed in Fig. 8(a). The absorber’s metamaterial nature and its capacity to control electromagnetic waves in novel ways are confirmed by the real part of n showing negative values, especially above 0.6 μm. The structure is well suited for uses including cloaking, optical filtering, and energy harvesting because of its negative refractive index, which shows that it facilitates negative refraction and wavefront control. The imaginary part of n, on the other hand, stays largely constant, indicating low energy loss and effective absorption over the operating wavelength range.

Figure 8(b) presents the impedance (Z) as a function of wavelength, highlighting its real and imaginary components. The real part of Z fluctuates between positive and negative values, while the imaginary part also varies, indicating strong interactions between incident electromagnetic waves and the absorber structure. Figure 8(c) illustrates the permittivity (ɛ) of the material, displaying both its real and imaginary components. The real part of ɛ exhibits negative values across multiple wavelength regions, particularly within the visible to near-infrared spectrum (0.4–1.4 μm). Figure 8(d) presents the analysis of permeability (µ), revealing that its real part exhibits negative values across multiple wavelength bands. This negative permeability (MNG mode) behaviour confirms that the absorber functions as a magnetic metamaterial. Figure 8(e) presents a comparative analysis of the calculated and simulated absorption spectra, providing validation for the absorber’s performance. The strong correlation between these two datasets confirms the accuracy of the computational model, reinforcing the absorber’s effectiveness in capturing and confining incident energy across the studied wavelength range. The simulated absorption exhibits distinct peaks at 0.4 μm, 0.75 μm, 1.1 μm, and 1.4 μm, demonstrating that Design M2 supports multi-wavelength selective absorption rather than continuous broadband absorption.

## The effect of magnitude and electrical fields

**Fig. 9:**
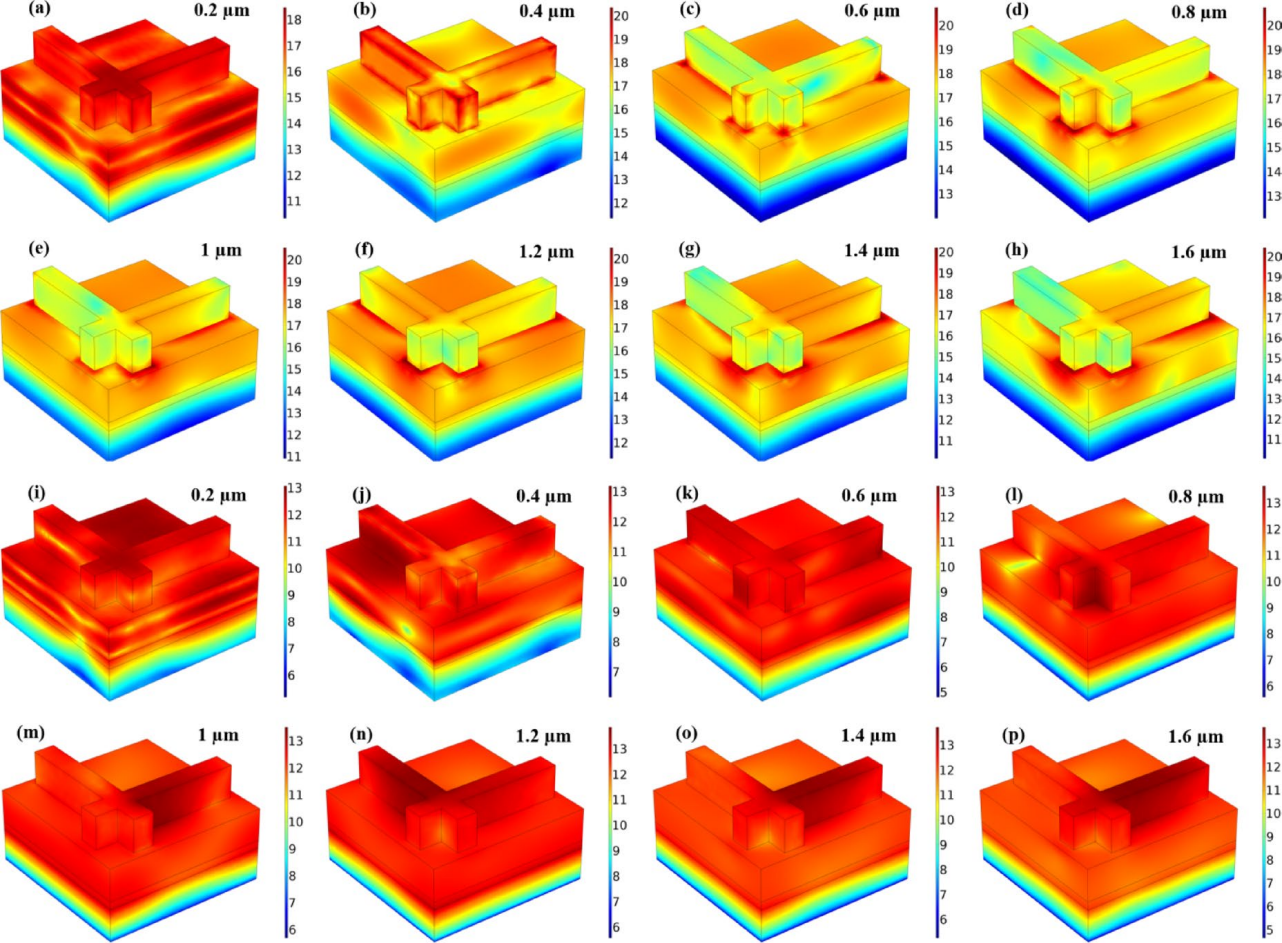
The variation in the normalised (**a**-**h**) electric field and magnetic field (**i**-**p**) in logarithmic scale for the M1 resonator-based structure.

In MIM absorber designs, the spatial distribution and magnitude of the electric field dictate the strength of resonant coupling and overall absorption efficiency. Understanding these electric field behaviours guides the optimisation of layer thicknesses, resonator geometry, and material selection to achieve broadband and angle-resilient absorption. Figure 9 presents the normalised electric field intensity (|E|) and the normalised magnetic field intensity (|H|) in logarithmic scale for different wavelengths (0.2 μm to 1.6 μm) in the Mode M1 resonator-based absorber structure. Figure 9(a-h) illustrates the normalised electric field intensity (|E|). The field distribution varies across the structure, demonstrating strong localisation of the electric field at resonant wavelengths. At shorter wavelengths (0.2 μm and 0.4 μm, Figures (a and b)), intense field confinement is observed around the top resonator edges, indicating strong plasmonic excitation. As the wavelength increases (0.6 μm to 1.2 μm, Figures (c-f)), the field gradually shifts towards the dielectric and metal interface, showing enhanced energy coupling within the absorber. For longer wavelengths (1.4 μm and 1.6 μm, Figures (g and h)), the field intensity weakens and spreads over a broader region, suggesting reduced confinement and lower absorption efficiency. These results confirm the absorber’s ability to efficiently trap and confine electromagnetic energy at specific resonances, making it suitable for optical sensing, photonic filtering, and energy harvesting applications. The Fig. 9(i-p), illustrates the normalised magnetic field intensity (|H|) in logarithmic scale at various wavelengths (0.2 μm to 1.6 μm) for the Mode M1 resonator-based absorber structure, complementing the electric field distribution analysis. Unlike the electric field (|E|), which exhibits strong confinement near the top resonator edges, the magnetic field intensity is more evenly distributed across the metal-dielectric interfaces, demonstrating magnetically induced resonance effects. At shorter wavelengths (0.2 μm and 0.4 μm, Figures (i and j)), localised magnetic field enhancement is observed at the top resonator arms and bottom metal layer, indicating strong plasmonic coupling. As the wavelength increases (0.6 μm to 1.2 μm, Figures (k-n)), the field penetrates deeper into the dielectric layers, suggesting enhanced energy storage within the structure. At longer wavelengths (1.4 μm and 1.6 μm, Figures (o and p)), the magnetic field intensity weakens, confirming a lower resonance effect at these spectral points. The results reinforce that the absorber achieves efficient energy confinement through both electric and magnetic resonances, making it highly effective for broadband absorption, electromagnetic wave manipulation, and metamaterial-based photonic applications.

**Fig. 10:**
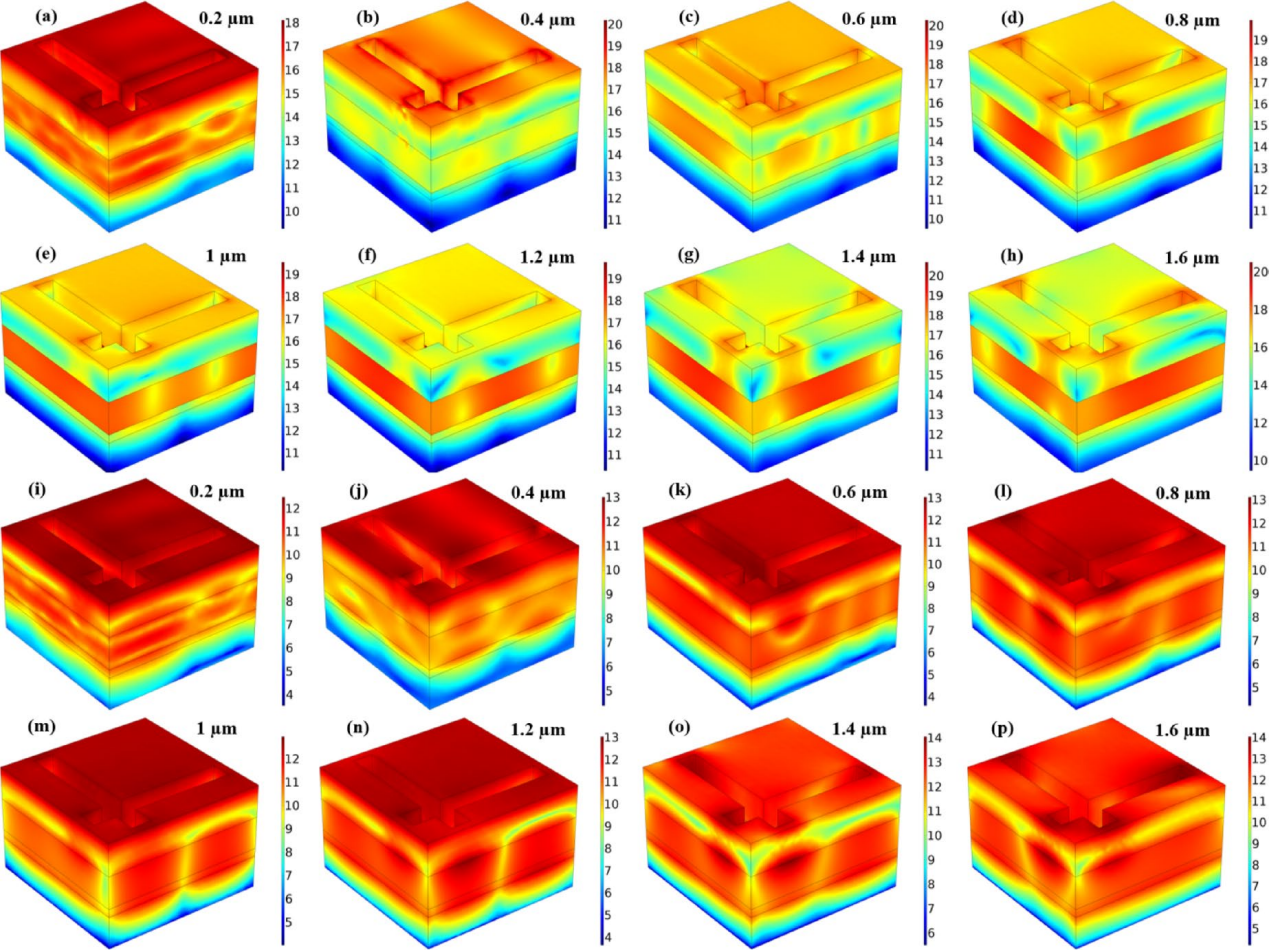
The variation in the normalised (**a**-**h**) electric field and magnetic field (**i**-**p**) in logarithmic scale for the M2 resonator-based structure.

The Fig. 10, illustrates the normalized electric field intensity (|E|) and the normalized magnetic field intensity (|H|) in logarithmic scale for different wavelengths (0.2 μm to 1.6 μm) in the Mode M2 (complementary L-shaped resonator) absorber structure, highlighting the distinct field confinement behavior compared to Mode M1. Unlike Mode M1, where the electric field is strongly localised around the resonator edges, from Fig. 10(a-h), we can see that mode M2 exhibits a more distributed field pattern, with energy coupling extending deeper into the dielectric and bottom metal layers. At shorter wavelengths (0.2 μm to 0.6 μm, Figures (a-c)), the field is concentrated near the resonator aperture regions, indicating enhanced localised plasmonic interactions. As the wavelength increases (0.8 μm to 1.2 μm, Figures (d-f)), the field intensity shifts toward the metal-dielectric interfaces, suggesting stronger cavity resonance effects. At longer wavelengths (1.4 μm to 1.6 μm, Figures (g and h), the field distribution becomes more uniform across the entire structure, confirming weaker resonance coupling. These results indicate that Mode M2 supports multi-wavelength resonance behaviour, making it highly suitable for wavelength-selective photodetection, filtering, and energy absorption applications requiring tunable spectral response. The Fig. 10(i-p) presents the normalised magnetic field intensity (|H|) in logarithmic scale at different wavelengths (0.2 μm to 1.6 μm) for the Mode M2 (complementary L-shaped resonator) absorber structure, further complementing the electric field analysis. Compared to Mode M1, where the magnetic field was concentrated near the resonator edges and interfaces, Mode M2 exhibits a more dispersed field distribution, with stronger field enhancement near the substrate and resonator boundaries. At shorter wavelengths (0.2 μm to 0.6 μm, Figures (i-k)), high-intensity magnetic fields are observed in the resonator gap and bottom metal layer, indicating localised magnetic dipole excitation. As the wavelength increases (0.8 μm to 1.2 μm, Figures (l-n)), the field intensity becomes more evenly distributed, suggesting enhanced resonant energy trapping within the absorber layers. At longer wavelengths (1.4 μm to 1.6 μm, Figures (o and p)), the magnetic field forms distinct patterns along the layered structure, confirming multi-wavelength resonance behaviour and deep field penetration into the bottom substrate. This variation highlights the strong coupling between magnetic and electric resonances in Mode M2, making it highly effective for polarisation-sensitive photonic applications, selective energy absorption, and magnetic field-enhanced optical devices.

## Fabrication methods

There are various technical obstacles to overcome before the suggested multi-layered solar absorber designs can be used in practical applications. Assuring accurate layer deposition and choosing the best fabrication method, like Chemical Vapour Deposition (CVD)^39,40^, to preserve compatibility with the desired application, are two of the fundamental challenges. Furthermore, creating nanoscale resonator structures is extremely difficult and calls for sophisticated techniques like Electron Beam Lithography (EBL) for highly accurate patterning^41,42^. The choice of materials is crucial to the success of these fabrication procedures because they must provide ideal optical qualities and structural stability while blending in perfectly with the manufacturing processes. Another important consideration is scalability, since moving from lab-scale fabrication to large-scale commercial production necessitates economical and effective manufacturing techniques^43^. High-performance solar absorbers can be implemented more easily in the actual world if creative fabrication techniques resolve these issues. In this work, a unique selective solar absorber design that effectively converts sunlight into heat using semiconductor-based metamaterials is presented. The careful selection and placement of materials constitutes the fundamental innovation. To attain the intended optical responses, the design combines explicitly silicon dioxide (SiO₂) layers with gold (Au) patterned in both pulse and circular geometries. High-precision fabrication is ensured by using EBL, which also supports environmental sustainability and manageable production costs. By adding a TI layer, the absorbing architecture is further improved. By reducing energy losses at material interfaces and increasing charge transport efficiency, these materials—which are renowned for their conductive surfaces and insulating interiors—improve light control. This layer enhances optical performance and adds structural robustness. It is made with methods such as Molecular Beam Epitaxy (MBE) to guarantee high crystalline quality and smooth integration. Next-generation solar cells with enhanced light absorption and energy conversion capabilities are made possible by this breakthrough. Metamaterials offer remarkable control over light absorption in contrast to traditional materials. Researchers may precisely control their optical properties to enhance energy collection by carefully constructing their thickness, composition, and structure. Because of their versatility, metamaterials hold great promise for creating solar energy harvesting systems of the future that are more effective and efficient. Table 2 compares the suggested design with earlier research that has been published. Comparative analysis is considered for the different parameters, including the wavelength of operation, maximum absorption amplitude, and the material employed for structure creation.

**Table 2:** Comparative analysis of the proposed structure with related solar cell materials.

Ref.	Materials	Wavelength ($\:{\upmu\:}\text{m}$)	Maximum Absorption Amplitude
This Study	Au, Si/SiO_2_/InP, TI (Bi_1.5_Sb_0.5_Te_1.8_Se_1.2_)	$\:0.2-1.6$	> 99.9%
^44^	Si, SiO_2_, Ti,	$\:0.3-2.5$	99%
^45^	SiO_2_, Tungsten, Si_3_N_4_	$\:0.44-0.54$	99.8%
^46^	Ni/SiO_2_	$\:0.4-0.7$	99%
^47^	Au/MgF2, Tungsten	$\:0.25-3$	> 98%
^48^	ZnSe, Cu	$\:8.5-20$	99.28%
^49^	Au, Cu, Ag, Al	$\:0.4-0.66$	99.7%
^50^	Al, Ga As	$\:0.44-0.6$	99.96%
^51^	W, AlN	$\:1-4$	41%
^52^	Au, SiO2	$\:0.7-1.9$	97.88%
^53^	TiN, SiO2, TiO2	$\:0.19-1.9$	> 90%
^54^	W/MgF2, Ti	$\:0.3-20$	78%
^55^	Au, SiO_2_	$\:0.5-2.5$	~ 90%
^56^	Glass, Ag, SiO_2_	$\:0.3-0.75$	92%
^57^	graphene/dielectric/graphene	$\:1.5-4.5$ THz	$\:-$
^58^	Ag, SiO_2_	$\:0.5-0.8$	$\:-$
^59^	Au, graphene, SiO_2_	$\:0.5-2.5$ THz	98.9%
^38^	Metal, TI, SiO_2_	$\:0.2-1.6$	close to 1 or 100%
^60^	Si3N4-TiN, SiO_2_, Ti	$\:280-2500\:\text{n}\text{m}$	97.77%
^61^	TiN,	$\:300-2500\:\text{n}\text{m}$	95.2%
^62^	graphene SiO_2_, Au	$\:26.73995\:\text{T}\text{H}\text{z}\:\text{a}\text{n}\text{d}\:26.75145\:\text{T}\text{H}\text{z}$	99.72 and 99.64%

## Conclusion

We developed an advanced solar absorber with a layered architecture that integrates dual-shaped metal resonators, designed to maximise light absorption efficiency. This innovative structure combines a mix of materials, including metals (Fe, Ti, Cu, Zn, Ag, Au), insulators (Si, SiO₂, InP), and a TI (Bi₁.₅Sb₀.₅Te₁.₈Se₁.₂), ensuring optimal interaction with incoming light. Using COMSOL Multiphysics simulations, we optimised the absorber’s performance across a broad wavelength range (0.2 to 1.6 μm), analysing the impact of layer thickness, unit cell dimensions, and resonator orientation on absorption efficiency. Our results demonstrate that the proposed design achieves over 90% absorption across the spectrum, making it highly effective for solar energy harvesting. Compared to existing absorbers operating in the UV, visible, and IR regions, our design offers superior wavelength coverage, capturing a broader spectrum of sunlight. Additionally, the adjustable resonator sizes provide greater tuning flexibility, allowing for enhanced performance beyond that of traditional wideband absorbers. This breakthrough could lead to the development of more efficient solar cell systems, particularly by integrating parasitic absorbers that harness otherwise unused sunlight.

## Data Availability

All data generated or analyzed during this study are included in this article.
